# The Lactate–Mitokine Axis in Exercise Adaptation: Linking Glycolytic and Mitochondrial Stress Signaling

**DOI:** 10.3390/ijms27219871

**Published:** 2026-11-04

**Authors:** Amelia Tero-Vescan, Ruxandra Ștefănescu, Bianca Eugenia Ősz, Mădălina Buț, Amalia Pușcaș, Mark Slevin

**Affiliations:** 1 Biochemistry Department, George Emil Palade University of Medicine, Pharmacy, Science, and Technology of Târgu Mureș, 38th Gh. Marinescu Street, 540139 Târgu Mureş, Romania; amelia.tero-vescan@umfst.ro (A.T.-V.); madalina.but@umfst.ro (M.B.); amalia.puscas@umfst.ro (A.P.); 2 Pharmacognosy and Phytotherapy Department, George Emil Palade University of Medicine, Pharmacy, Science, and Technology of Târgu Mureș, 38th Gh. Marinescu Street, 540139 Târgu Mureş, Romania; 3 Pharmacology and Clinical Pharmacy Department, George Emil Palade University of Medicine, Pharmacy, Science, and Technology of Târgu Mureș, 38th Gh. Marinescu Street, 540139 Târgu Mureş, Romania; bianca.osz@umfst.ro; 4 Center for Advanced Medical and Pharmaceutical Research, George Emil Palade University of Medicine, Pharmacy, Science, and Technology of Târgu Mureș, 38th Gh. Marinescu Street, 540139 Târgu Mureş, Romania; mark.slevin@umfst.ro

**Keywords:** lactate, mitokines, exercise, mitochondrial stress, FGF21, GDF15, MOTS-c, humanin, mitochondrial adaptation

## Abstract

Exercise induces coordinated metabolic and mitochondrial stress responses that extend beyond ATP production to regulate cellular and systemic adaptation. Lactate, traditionally viewed as a glycolytic product, is now recognized as an oxidative substrate, interorgan carbon shuttle, and signaling metabolite, whereas mitochondrial stress promotes adaptive signaling and the release of mitokines and mitochondrial-derived peptides. This narrative review proposes the lactate–mitokine axis as a conceptual framework integrating these two exercise-responsive systems, while distinguishing experimentally established mechanisms from potential interactions that remain to be functionally validated. Repeated exercise promotes mitochondrial remodeling and increases oxidative capacity, which in turn reshapes the lactate response to subsequent exercise, as reflected by lower post-exercise lactate concentrations and enhanced lactate clearance after training. Conversely, increased lactate flux may influence mitochondrial adaptation through metabolic, redox, and receptor-dependent signaling, supporting a potentially bidirectional relationship between lactate handling and mitochondrial remodeling. The relative contribution of these interconnected responses appears exercise modality-dependent, with high-intensity, prolonged endurance, and resistance exercise generating distinct combinations of glycolytic and mitochondrial stress. Although lactate metabolism and mitochondrial stress signaling share several regulatory pathways, direct causal interactions between lactate signaling and mitokine expression or secretion during exercise remain insufficiently established. Thus, the proposed lactate–mitokine axis should be regarded as a hypothesis-generating framework requiring further experimental validation.

## 1. Introduction

Physical exercise is no longer considered simply a muscle contraction involving ATP consumption and the coordinated mobilization of energy substrates, oxygen transport to the cells, and mitochondrial activation. The transient changes occurring during or after training trigger a cascade of molecular programs involving metabolic, transcriptional, inflammatory, vascular, and endocrine pathways [1]. Recent multi-omic studies such as that of the Molecular Transducers of Physical Activity Consortium (MoTrPAC) have shown that acute cardiopulmonary exercise produces thousands of temporally coordinated changes across the metabolome, lipidome, proteome, transcriptome, and immunome, involving pathways related to energy metabolism, oxidative stress, inflammation, tissue repair, and growth factor signaling [2]. These changes are in fact a source of biologically active exerkines that facilitate communication between muscle, adipose tissue, and other organs [3].

One such example is lactate, long considered a metabolic end product, but which is now recognized as an oxidative substrate, an intercellular and interorgan carbon shuttle, and a signaling metabolite capable of activating receptor-dependent pathways and influencing cellular adaptation [4].

In addition to producing ATP, mitochondria trigger stress-responsive signaling by producing molecules with endocrine effects—such as fibroblast growth factor 21 (FGF21) and growth differentiation factor 15 (GDF15), as well as mitochondrial-derived peptides like mitochondrial ORF of the 12S rRNA type-c (MOTS-c)—which play a role in regulating metabolism and muscle performance [5,6].

Recent studies have substantially changed our understanding of exercise-induced metabolic and mitochondrial signaling. Among the emerging mechanisms, protein lactylation has gained attention as a potential regulator of exercise adaptation through epigenetic modifications of histones and post-translational modifications of non-histone proteins, including metabolic enzymes. However, exercise-induced changes in protein lactylation reported in several animal models have not been consistently confirmed in human skeletal muscle, highlighting important gaps in the current evidence [7]. Beyond intracellular metabolic regulation, exercise-responsive factors released by skeletal muscle, including myokines, metabolites, and mitochondrial-derived peptides, have been investigated as mediators of interorgan communication and systemic metabolic adaptations, particularly in the context of aging and obesity [8]. Furthermore, mitochondrial stress-induced adaptive responses, collectively associated with mitohormesis, involve the activation of the integrated stress response and mitochondrial unfolded protein response (UPRmt), together with the production of stress-responsive factors such as FGF21, GDF15, and MOTS-c [9].

However, an important mechanistic question remains unanswered: whether exercise-induced lactate signaling directly influences endocrine pathways triggered by mitochondrial stress, or whether changes in lactate and mitokines operate in parallel and independently during exercise. Although these two signaling pathways converge on common regulatory mechanisms, such as AMPK, redox homeostasis, and PGC-1α, this does not necessarily demonstrate the existence of a direct regulatory interaction. Furthermore, the extent to which lactate availability influences the expression or secretion of stress-response factors such as FGF21, GDF15, MOTS-c, and Humanin during exercise remains insufficiently elucidated.

The scientific rationale behind the potential lactate–mitokine relationship is based on the metabolic interdependence between glycolytic activity and mitochondrial function during exercise. Lactate production and oxidation influence cellular carbon flux and redox balance, whereas mitochondrial energetic stress activates adaptive pathways that regulate metabolic remodeling and stress-responsive signaling. These processes converge on regulatory mechanisms, providing a plausible mechanistic basis for their functional interaction. However, such convergence may reflect parallel responses to exercise-induced metabolic stress rather than direct communication between lactate-dependent signaling and mitokine regulation. Emerging experimental evidence, particularly regarding lactate-mediated regulation of FGF21, supports functional interactions between these systems, although their molecular mechanisms, tissue specificity, and relevance across different exercise conditions remain incompletely understood. Further investigations are therefore needed to establish the extent and physiological significance of these interactions and to determine whether similar regulatory relationships extend to other mitokines. In this context, this review aims to critically evaluate the concept of the lactate–mitokine axis as a potential bidirectional mechanism linking glycolytic flux, lactate-dependent signaling, mitochondrial stress responses, and endocrine adaptations. Without assuming a direct causal relationship, this article distinguishes experimentally demonstrated interactions from hypothetical mechanisms. It critically analyzes evidence from experimental models, animal studies, and, where available, human studies, while examining how the type, intensity, and duration of exercise, as well as training status, can influence these responses. Particular attention is paid to distinguishing between acute molecular responses induced by exercise and chronic adaptations associated with training, as well as to identifying the experimental approaches needed to determine whether lactate directly regulates endocrine factors associated with mitochondrial stress or whether both pathways are activated in parallel in response to a common metabolic stress.

## 2. Literature Search Methodology

A narrative literature search was conducted using PubMed/MEDLINE, Scopus, and Web of Science to identify publications addressing lactate metabolism, mitochondrial stress signaling, and exercise-induced adaptations. Search terms included combinations of “lactate”, “lactate signaling”, “lactate shuttle”, “lactylation”, “mitochondrial stress”, “mitohormesis”, “mitokines”, “mitochondrial-derived peptides”, “FGF21”, “GDF15”, “MOTS-c”, “Humanin”, “exercise”, and “exercise adaptation”. Priority was given to studies published within the last five years (2021–2026), while earlier landmark studies were included when necessary to establish fundamental biochemical mechanisms or provide historical context.

The literature selection primarily focused on original experimental studies involving human participants, animal models, and cellular systems that investigated the molecular mechanisms linking glycolytic activity, lactate signaling, mitochondrial adaptation, and stress-responsive endocrine factors. Attention was paid to studies examining direct mechanistic interactions, including experimental manipulation of lactate availability, transport, or receptor-mediated signaling. Relevant review articles were also consulted to provide conceptual context and identify additional primary publications.

The selected literature was critically evaluated according to experimental relevance, study design, and mechanistic strength, distinguishing direct experimental evidence from associative findings and proposed regulatory interactions. As this article represents a narrative rather than a systematic review, no formal meta-analysis or quantitative synthesis was performed.

## 3. The Lactate Paradox: From Metabolic End Product to Exercise Signal

### 3.1. Lactate Production During Exercise: Revisiting the Classical Paradigm

**Figure 1:**
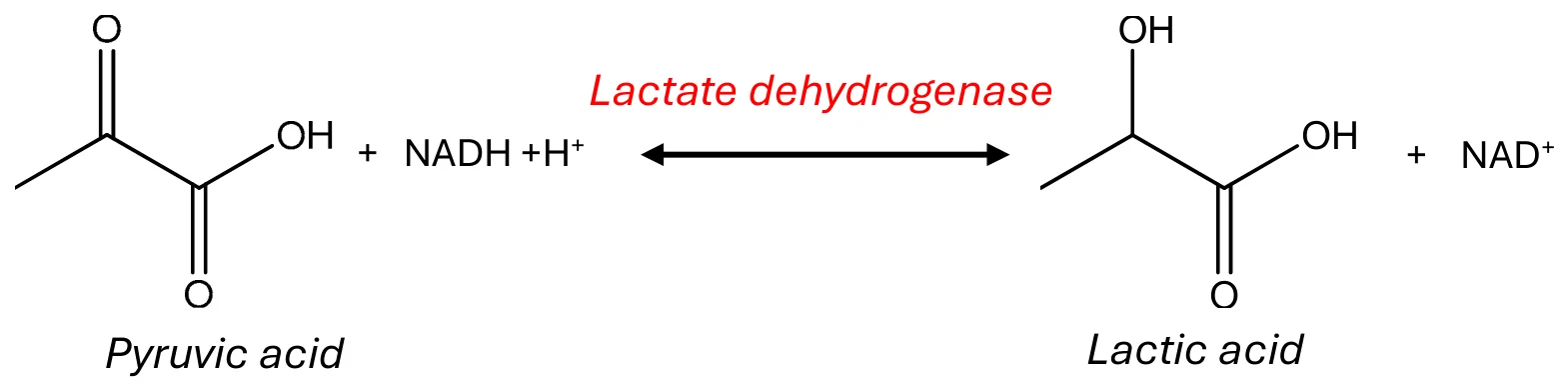
Reaction catalyzed by lactate dehyrogenase.

Lactate is produced during both aerobic and anaerobic exercise as glycolytic flux increases. Pyruvate can either undergo mitochondrial oxidation or be reversibly converted to lactate by lactate dehydrogenase (LDH), depending on cellular metabolic demands (Figure 1) [10].

The conversion of pyruvate to lactate oxidizes NADH to NAD^+^, thereby maintaining the availability of the NAD^+^ required for the glycolysis reaction catalyzed by glyceraldehyde-3-phosphate dehydrogenase (GA3PDH); lactate production thus serves as a mechanism supporting glycolysis at high rates [11]. NADH produced during glycolysis can follow two major metabolic pathways: the transfer of reducing equivalents into the mitochondria to enter the electron transport chain via the malate–aspartate and glycerol-3-phosphate shuttles, or reoxidation mediated by LDH.

**Figure 2:**
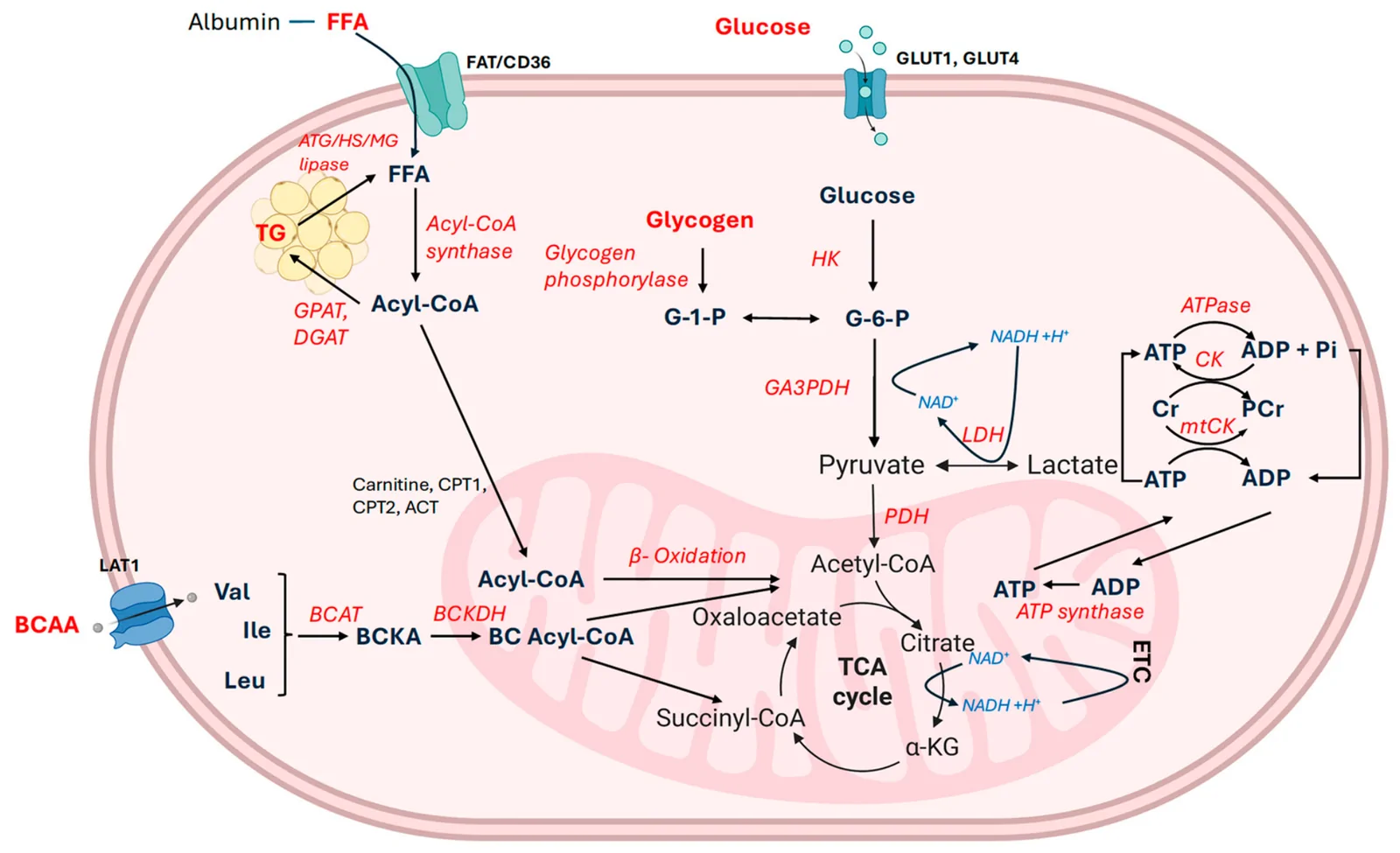
Integrated energy substrate metabolism in skeletal muscle during exercise. Abbreviations: ACT—acylcarnitine translocase; ADP—adenosine diphosphate; α-KG—α-ketoglutarate; ATP—adenosine triphosphate; ATPase—adenosine triphosphatase; BCAA—branched-chain amino acids; BCAT—branched-chain amino acid aminotransferase; BCKA—branched-chain α-keto acid; BCKDH—branched-chain α-keto acid dehydrogenase; CK—creatine kinase; CPT1—carnitine palmitoyltransferase 1; CPT2—carnitine palmitoyltransferase 2; Cr—creatine; DGAT—diacylglycerol acyltransferase; ETC—electron transport chain; FAT/CD36—fatty acid translocase/cluster of differentiation 36; FFA—free fatty acids; G-1-P—glucose-1-phosphate; G-6-P—glucose-6-phosphate; GA3PDH—glyceraldehyde-3-phosphate dehydrogenase; GLUT1—glucose transporter 1; GLUT4—glucose transporter 4; GPAT—glycerol-3-phosphate acyltransferase; HK—hexokinase; HS—hormone-sensitive lipase; Ile—isoleucine; LAT1—L-type Amino Acid Transporter 1; LDH—lactate dehydrogenase; Leu—leucine; MCT—monocarboxylate transporter; MG lipase—monoglyceride lipase; mtCK—mitochondrial creatine kinase; NAD^+^—nicotinamide adenine dinucleotide; NADH + H^+^—reduced nicotinamide adenine dinucleotide; PCr—phosphocreatine; PDH—pyruvate dehydrogenase; Pi—inorganic phosphate; TCA—tricarboxylic acid; TG—triglycerides; Val—valine.

The reversible LDH reaction links lactate–pyruvate interconversion to cellular NADH/NAD^+^ balance, supporting metabolic and redox communication [9]. The major pathways of skeletal muscle substrate utilization and ATP production are summarized in Figure 2 [12].

Circulating lactate is a dynamic parameter, an equilibrium between the amount produced at a given time and the amount utilized for energy by the heart or in hepatic and renal gluconeogenesis. The amount of lactate produced depends on exercise intensity, duration, nutritional status, muscle-fiber recruitment, and training status. Thus, endurance training increases mitochondrial oxidative capacity and alters the expression of monocarboxylate transporters (MCT) involved in lactate exchange, thereby improving the capacity for lactate redistribution and utilization [13].

The lactate G protein-coupled receptor 81 (GPR81)/hydroxycarboxylic acid receptor 1 (HCAR1) is 2.5 times more abundant in type II glycolytic fibers than in type I oxidative fibers. Skeletal muscle biopsies obtained from healthy men and women following leg-extension exercise showed, most significantly, a 25% higher GPR81 expression in women, an overall increase in protein kinase A (PKA), a decrease in cyclic adenosine monophosphate response element-binding protein (CREB-S133) phosphorylation, and increased expression of vascular endothelial growth factor (VEGF) and peroxisome proliferator-activated receptor gamma coactivator 1-alpha (PGC-1α) [14].

Twice-daily high-intensity interval exercise (HIIE) increased skeletal muscle MCT1 gene expression immediately and 3 h post-exercise, whereas MCT4 levels remained unchanged. After 3 weeks of training, the acute increase in MCT1 did not translate into a differential chronic blood lactate response; consequently, participants reached a higher exercise intensity before attaining the same blood lactate level [15].

Moreover, exogenous lactate administration to sedentary animals for four weeks induced metabolic changes, such as an increase in mitochondrial carbohydrate metabolism through the activation of citrate synthase and malate dehydrogenase 2 [16].

These findings implicate lactate as a versatile molecule that, depending on metabolic demand, functions as a fuel, an interorgan carbon shuttle, and an exercise-responsive signal capable of linking glycolytic activity to broader cellular adaptive responses. This signaling dimension is particularly relevant during exercise, when increased lactate flux occurs alongside mitochondrial energetic and redox stress, creating the potential for convergence between lactate-dependent signaling and mitochondrial stress-responsive pathways.

### 3.2. Lactate Threshold and Exercise Intensity

**Figure 3:**
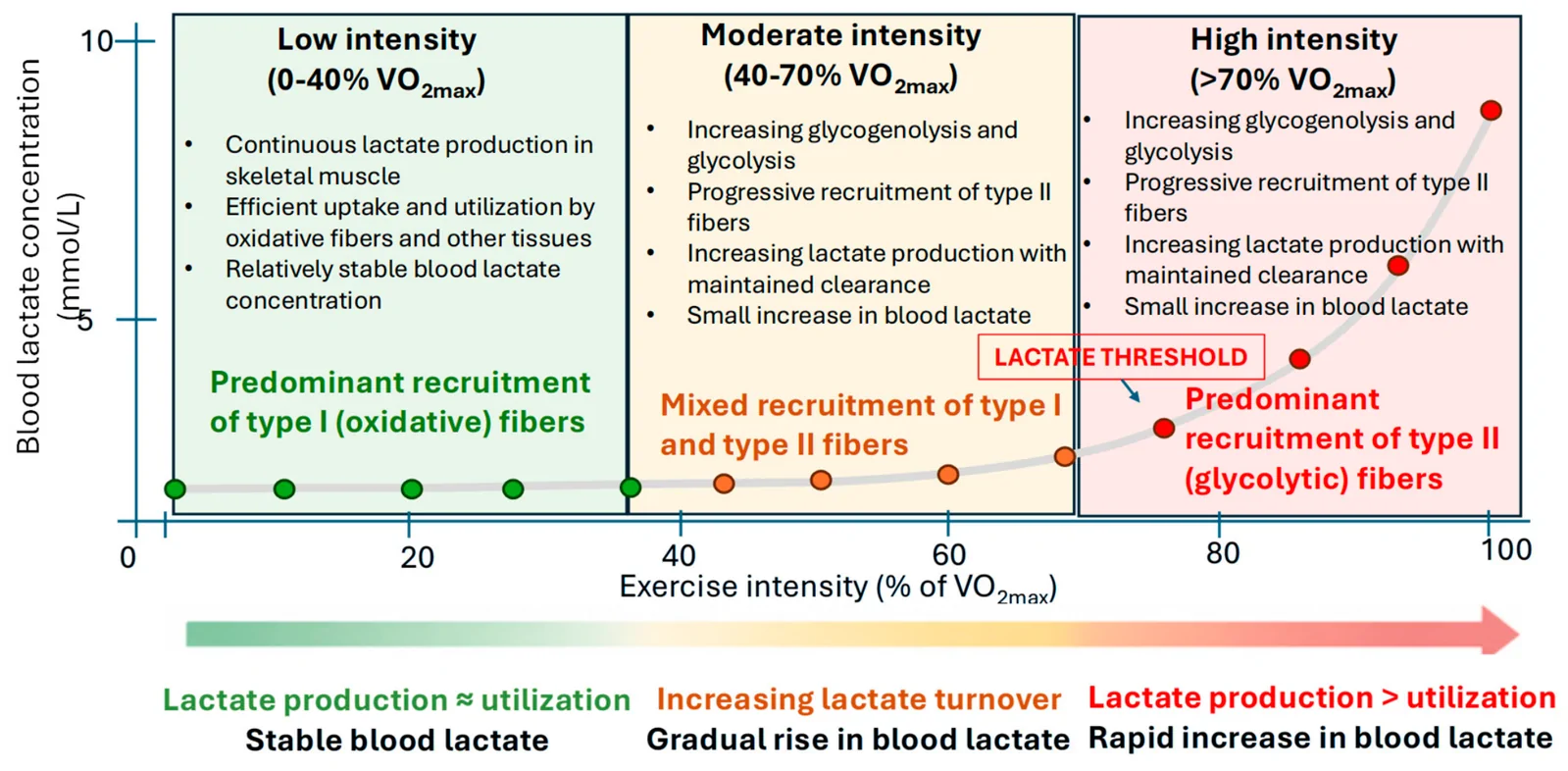
Exercise intensity-dependent changes in skeletal muscle fiber recruitment and lactate turnover. Note: The exercise intensity ranges expressed as percentages of VO_2_max are illustrative rather than fixed physiological boundaries. The corresponding lactate responses and metabolic transitions vary according to training status, exercise protocol, and individual physiological characteristics. LT does not delineate the switch from aerobic to anaerobic metabolism; in fact, it more accurately predicts the shift in the equilibrium concentration between the amount of lactate produced during exercise and its clearance by cardiac utilization and gluconeogenesis. The LT value exhibits inter-individual variability, with lactate homeostasis being highly sensitive around this point; for instance, increasing swimming speed by just 0.01 m·s^−1^ near the LT resulted in lactate accumulation, whereas reducing speed by 0.010.015 m·s^−1^ substantially decreased such accumulation [18].

Blood lactate concentration increases nonlinearly with exercise intensity. At low to moderate intensities, lactate production is balanced by its uptake and utilization, whereas higher intensities increase glycolytic flux and type II muscle fiber recruitment, eventually exceeding lactate clearance and causing a sharp rise in blood lactate, referred to as the lactate threshold (LT) (Figure 3) [17]. The relative exercise intensity at which lactate accumulation becomes pronounced is not universal, as it is influenced by training status, exercise modality, recruitment patterns, and individual metabolic characteristics.

Although LT and maximal lactate steady state (MLSS) are closely related, they represent physiologically distinct concepts. LT is identified during incremental exercise as a change in the blood lactate response to increasing workload, with its determination depending on the specific threshold definition and analytical method employed. In contrast, MLSS represents the highest constant exercise intensity at which blood lactate concentration remains approximately stable over time, reflecting a dynamic balance between lactate synthesis and consumption. These parameters are not interchangeable, and their correspondence depends on the method used to identify LT. Determination of MLSS during 30 min constant-speed running tests revealed substantial interindividual variability, with blood lactate concentrations ranging from 2.9 to 6.5 mmol·L^−1^ [19], whereas in recreational cyclists, this value was 5.43 ± 1.57 mmol·L^−1^ during 30 min of constant-load exercise [20].

Physical effort at intensities above the LT, such as during high-intensity interval training (HIIT), leads to a substantial rise in blood lactate concentration; this does not signify metabolic stagnation, but rather an accelerated glycolytic flux. Exercise performed 0.5 km·h^−1^ above the MLSS resulted in progressive blood lactate accumulation, while pulmonary VO_2_ remained stable. Consequently, blood lactate kinetics and the whole-body oxidative steady state can become uncoupled, and blood lactate levels alone do not reflect the entire metabolic flux [21].

In summary, the LT is a dynamic value that varies between individuals. During exercise below the LT, plasma lactate concentration remains relatively constant because the rates of production and clearance are roughly balanced. At exercise intensities exceeding MLSS, lactate appearance progressively exceeds its disappearance, resulting in sustained blood lactate accumulation and the inability to maintain a lactate steady state.

### 3.3. From Lactate Exposure to Adaptive Signaling

Lactate serves as a marker of both metabolic flux and adaptation to exercise. Upon repeated oral administration, lactate modulated mitochondrial adaptations are associated with endurance training in mice by increasing cytochrome c oxidase activity (COX) in the oxidative soleus muscle, as well as in cardiac muscles. However, lactate did not influence adenosine monophosphate-activated protein kinase (AMPK), acetyl-CoA carboxylase (ACC), p38 mitogen-activated protein kinase (p38 MAPK), or calcium/calmodulin-dependent protein kinase I (CaMKII) signaling. In addition, it failed to induce MCT remodeling in a manner like physical exercise; this suggests it acts as a selective modulator of specific pathways involved in exercise adaptation rather than as a substitute for exercise itself [22].

The signaling effects of lactate are not limited to mitochondrial metabolism. Intraperitoneal administration of lactate (1 g/kg) for two weeks to calorie-restricted mice increased the activity of the mitochondrial enzymes citrate synthase and succinate dehydrogenase and the expression of several oxidative phosphorylation (OXPHOS) proteins. Concomitant attenuation of the reduction in myofiber cross-sectional area and preservation of grip strength were noted outcomes [23].

However, exogenous administration of lactate did not further enhance exercise-induced mechanistic target of rapamycin complex 1 (mTORC1)-associated signaling or muscle protein synthesis in mice subjected to acute electrically stimulated high-intensity contractions or two weeks of mechanical overload [24]. Intravenous administration of sodium lactate to human subjects during one-legged resistance exercise, although increasing circulating lactate from approximately 3 to 7 mmol·L^−1^, did not further alter the exercise-induced activation of mechanistic target of rapamycin (mTOR), ribosomal protein S6 kinase 1 (S6K1), and extracellular signal-regulated kinase (ERK) signaling [25].

Experimental lactate administration provides valuable mechanistic insights into the potential regulatory effects of lactate on mitochondrial metabolism. However, these findings should be distinguished from responses to endogenous lactate production during exercise, where lactate dynamics are accompanied by muscle contraction, changes in cellular energy demand, calcium signaling, and broader metabolic adaptations. Differences in lactate concentration, exposure duration, and tissue distribution may further influence the resulting biological responses. Therefore, mitochondrial adaptations observed following exogenous lactate administration support the signaling potential of lactate but do not independently establish its causal contribution to exercise-induced mitokine expression or secretion. These experimental models provide a useful basis for investigating whether lactate-dependent mechanisms contribute to the coordinated metabolic and mitochondrial stress responses elicited by exercise.

Protein lactylation is one of the mechanisms hypothesized to underline the role of lactate in long-term adaptation to exercise; however, experimental evidence is limited, and numerous sampling timepoints as well as a lactylomics approach are required. Although protein lactylation has been described in obese and insulin-resistant individuals [26], neither acute resistance exercise nor ten weeks of resistance training have demonstrated nuclear or cytoplasmic protein lactylation [27].

Future studies are needed to elucidate the mechanism by which lactate functions as a signaling molecule, as its effects are strongly influenced by the concentration, duration, and cellular location of lactate exposure, muscle phenotype, metabolic state, and accompanying contractile stress.

### 3.4. The Lactate Shuttle Concept in Modern Exercise Physiology

The lactate shuttle is particularly relevant during exercise because type II glycolytic fibers produce substantial amounts of lactate and express MCT4, which is involved in exporting lactate from the cell. Oxidative muscle fibers and other tissues, such as the heart, brain, liver, and kidneys, express MCT1, which plays a role in lactate uptake [28].

Endurance training models the lactate shuttle towards a faster oxidative disposal as demonstrated using [3-^13^C]lactate and [6,6-^2^H_2_]glucose tracers in trained or untrained individuals. Experimentally increasing blood lactate availability through a lactate clamp increased lactate oxidation by ~75% and markedly reduced estimated muscle glycogen utilization, sparing endogenous carbohydrate stores [29]. The lactate shuttle also supplies a significant amount of energy to the heart, while the Cori cycle may allow for a glycogen resynthesis rate of approximately 1.9 mmol/kg/h during the first two hours following resistance training [30].

The lactate shuttle mechanism is not limited to physical exertion. Through the continuous administration of [3-^13^C]lactate and [6,6-^2^H_2_]glucose tracers to healthy individuals, both at rest and during an oral glucose tolerance test, it has been demonstrated that 38% of the 75 g glucose load passed through the circulating lactate pool over the two-hour observation period. Consequently, the lactate shuttle operates in a biphasic manner during both exercise and aerobic conditions, playing a role in cellular signaling via HCAR1/GPR81 and redox signaling [29].

### 3.5. MCT1 and MCT4 in Skeletal Muscle: Regulation of Lactate Flux and Exercise Adaptation

The transport of lactate together with H+ across the skeletal muscle sarcolemma occurs via MCT1/SLC16A1 and MCT4/SLC16A3 and is driven by proton and concentration gradients. Although both transporters can function bidirectionally, MCT1 is preferentially associated with oxidative muscle fibers and facilitates lactate uptake, whereas MCT4 is more abundant in glycolytic fibers and supports lactate efflux. Consequently, MCT1 is particularly responsive to training, whereas MCT4 shows a more variable and generally less consistent adaptive response [31].

The type of physical exertion influences the expression of skeletal muscle MCTs. In rats subjected to HIIT-style exercise for 8 weeks, an increase in MCT4, PGC-1α, and hypoxia-inducible factor 1-alpha (HIF-1α) mRNA expression was observed in both the oxidative soleus and the glycolytic extensor digitorum longus muscles, whereas MCT1 mRNA expression remained unchanged. However, energy-matched moderate-intensity continuous training (MICT) did not significantly alter MCT1, MCT4, or PGC-1α expression in either muscle [32].

Skeletal-muscle biopsies before, immediately after and 3 h after exercise in the case of acute HIIT-type effort showed that MCT1 mRNA increased immediately and remained elevated 3 h after HIIT, whereas MCT4 mRNA remained unchanged. Therefore, MCT1 and MCT4 regulation is isoform- and context-specific, depending on factors including exercise modality, training duration, metabolic state, and the time at which transporter expression is assessed [15]. By controlling the muscle fiber type where lactate is retained during physical exertion, MCT1 and MCT4 transporters indirectly determine the activation of mitochondrial metabolism in glycolytic fibers or redistribute lactate to oxidative muscle fibers.

### 3.6. HCAR1/GPR81 as a Lactate-Sensing Receptor

The cellular lactate receptor HCAR1 (also known as GPR81), a Gi protein-coupled membrane receptor that reduces cyclic adenosine monophosphate (cAMP) activity by inhibiting adenylyl cyclase, has been identified in skeletal muscle, the brain, and other tissues. HCAR1 distribution in skeletal muscle is fiber-type-specific. Analysis of muscle tissue biopsies from eight healthy men and eight women performing leg-extension exercises accompanied by intravenous sodium lactate infusion demonstrated that HCAR1 protein abundance was approximately 2.5-fold higher in type II glycolytic fibers than in type I oxidative fibers, and 25% greater in women than in men [14].

Lactate HCAR1 signaling is also present in tissues outside skeletal muscle. Five weeks of treadmill exercise increased hippocampal collagen IV expression, phosphoinositide 3-kinase (PI3K)/protein kinase B (Akt) and ERK1/2 signaling, and microvascular density in wild-type (WT) mice, whereas these exercise-induced responses were largely absent in HCAR1-deficient animals [33]. HCAR1 signaling in adipose tissue was demonstrated in diet-induced obese, insulin-resistant mice subjected to physical exercise. In this context, the activation of IRS1–Akt–GLUT4 signaling and GPR81 revealed that high-lactate exercise improved adipose and systemic insulin sensitivity [34].

All these studies indicate that the mechanism of action of lactate is tissue-dependent. While oxidative tissues can remove lactate from circulation and utilize carbon for energy production, HCAR1-expressing cells can potentially interpret the availability of extracellular lactate as a metabolic signal. These two processes can occur simultaneously: lactate uptake does not necessarily imply HCAR1 activation, and HCAR1 activation does not require lactate oxidation.

### 3.7. Lactylation as a Potential Metabolic–Epigenetic Link in Exercise Adaptation

Post-translational lactylation of lysine residues in histones and other types of proteins is one of the potential mechanisms influencing glycolysis, either by directly modifying the function of enzymes or indirectly through the process of transcription [7].

Data in the literature are contradictory. Some studies report that histone H3 lysine 9 lactylation (H3K9la) induced by exposure to 15 mM lactate for 3–5 days in C2C12 myoblasts and young mice activated the downstream *Neu2*-promoter and promoted myogenic differentiation [35]. Other studies attempting to replicate the physiological context of exercise suggest that lactate exposure is insufficient to produce biologically significant levels of lactylation. Acute resistance exercise, 10 weeks of resistance training, or a 60 min endurance cycling bout failed to alter global skeletal-muscle protein lactylation during the 8 h recovery period. C2C12 myotubes exposed to different sodium lactate concentrations and exposure durations showed no significant changes in nuclear or cytoplasmic protein lactylation [27].

The absence of detectable changes in global skeletal-muscle lactylation does not exclude transient, site-specific, or tissue-specific lactylation. Seven weeks of treadmill exercise or daily sodium L-lactate administration increased lactate availability and protein lactylation in ovariectomized mice and reduced the overall bone loss. This was attributed to H3K18 lactylation (H3K18la), which suppressed osteoclast differentiation and downregulated osteoclastogenic genes including *Ctsk*, *Mmp9*, and *Mmp12*, with alanyl-tRNA synthetase 1 (AARS1) and sirtuin 6 (SIRT6) as components regulating lactylation/delactylation [36].

Evidence from cellular and animal models suggests that lactylation may contribute to transcriptional regulation and longer-lasting metabolic adaptations. For example, H3K18 lactylation in experimentally trained human monocytes/macrophages persisted after withdrawal of the initial stimulus and was associated with enhanced expression of trained-immunity genes upon secondary stimulation. Pharmacological inhibition of lactate production or histone lactylation attenuated these responses [37]. However, these findings concern immune-cell reprogramming rather than exercise training and cannot be directly extrapolated to skeletal muscle adaptations. Importantly, studies in human skeletal muscle have not demonstrated significant changes in global protein lactylation following acute resistance exercise, prolonged resistance training, or endurance cycling [27].

Although lactylation represents a plausible mechanism linking metabolic activity to epigenetic regulation, its contribution to persistent exercise-induced adaptations in human skeletal muscle remains to be established. Future investigations employing site-specific lactylomics and extended post-exercise sampling are needed to clarify this potential relationship.

## 4. Exercise-Induced Mitochondrial Stress: From Bioenergetic Challenge to Signaling

### 4.1. ATP Turnover and Mitochondrial Energetic Stress

Skeletal muscle contraction dramatically increases the demand for ATP, which is consumed by myosin ATPases, Ca^2+^-ATPases involved in excitation–contraction, and Na^+^/K^+^-ATPase activity required to restore transmembrane ion gradients. Cellular ATP content cannot sustain such consumption; therefore, rapid alternative sources, such as creatine phosphate (PCr), high-energy compounds, and glycolysis are utilized. Consequently, the energetic stress generated by contraction is better reflected by changes in PCr, ADP, Pi, and the phosphorylation potential than by ATP concentration alone. The decrease in PCr concentration and the rise in ADP and Pi during contraction establish a feedback system through which ATP demand is signaled to the mitochondria [38].

Energy expenditure increases ADP concentration, which, via the adenylate kinase reaction (2 ADP ⇌ ATP + AMP), leads to the regeneration of a small amount of ATP while also producing AMP, which activates AMPK. Although AMP is a potent allosteric modulator of AMPK, ADP alone can modulate AMPK through the elevated concentrations observed during exercise. Consequently, the AMP/ATP ratio alone cannot fully explain AMPK activation across continuous and interval exercise conditions [39].

AMPKα2 activation depends on the individuals’ training status. Eleven untrained men were compared with seven endurance-trained cyclists and triathletes during 120 min of cycling at the same relative intensity of approximately 65% VO_2_peak. Despite exercising at nearly twice the absolute workload, trained participants exhibited a markedly smaller energetic disturbance. In untrained muscle, calculated free AMP increased approximately 17-fold. This greater energetic stress in untrained individuals was accompanied by a 370% increase in AMPKα2 activity, together with approximately twofold increases in AMPKα Thr172 and ACCβ Ser222 phosphorylation. In contrast, none of these AMPK signaling responses increased significantly in endurance-trained individuals. Thus, endurance training appears to attenuate the energetic perturbation generated by a given relative exercise intensity and can largely abolish AMPK activation during prolonged moderate-intensity exercise [40]. Repeated HIIT rapidly remodels skeletal-muscle mitochondrial function. In sedentary adults, seven HIIT sessions over 14 days, each comprising 10 × 1 min cycling intervals targeting ~90% of VO_2_peak, increased mitochondrial respiration per unit of mitochondrial protein by 23% with lipid-derived substrates, 18–22% through complex I, 14–19% through complexes I + II, and 24% through complex II. These functional adaptations occurred without increased whole-muscle respiratory-chain protein abundance and were accompanied by the transcriptional enrichment of mitochondrial respiratory pathways, particularly complex I, suggesting rapid improvements in mitochondrial protein quality [41].

Mitochondrial remodeling is non-stoichiometric; enzymes involved in the TCA cycle and fatty-acid oxidation are prioritized initially, whereas the amount of OXPHOS does not increase in proportion to mitochondrial mass. Normal-, high-, and reduced-volume HIIT protocols increased electron flow toward OXPHOS, a factor that appears far more important for enhancing ATP generation than simply increasing the abundance of respiratory-chain components [42].

Exercise-induced mitochondrial stress is generally transient and regulated, reflecting increased energetic demand, redox fluctuations, and activation of mitochondrial quality-control mechanisms that support adaptive remodeling. In contrast, mitochondrial dysfunction associated with pathological conditions or sustained experimental perturbations may involve persistent bioenergetic impairment, excessive oxidative stress, and compromised cellular homeostasis. Although both conditions can engage overlapping stress-responsive pathways, including AMPK, SIRT1, and PGC-1α-related signaling, their physiological consequences depend on the intensity, duration, and reversibility of the initiating stimulus. Thus, activation of mitochondrial stress signaling during exercise should not be equated with mitochondrial dysfunction.

Exercise promotes mitochondrial fission, evidenced by increased DRP1^Ser616 phosphorylation and cristae abnormalities within mitophagosome-like structures in the subsarcolemmal cleft, and even the presence of mitochondria in the extracellular space, potentially involving mitochondrial ejection [43].

### 4.2. Integration of Redox, Ca^2+^, and AMPK–SIRT1–PGC-1α Signaling in Mitochondrial Adaptation

Mitochondrial adaptation to exercise involves the integration of multiple signaling pathways. Increased ATP consumption and repeated excitation–contraction cycles alter cytosolic Ca^2+^ levels, while the elevated rate of oxidative reactions and oxidative phosphorylation increase reactive oxygen species (ROS) production. All these mechanisms lead to the activation of AMPK, CaMKII, p38 MAPK, NAD^+^-dependent SIRT1 signaling, and the transcriptional coactivator PGC-1α.

ROS are an important component of exercise-induced signaling; however, although their levels rise during exercise, not all originate from mitochondria (mtROS). In recreationally active young men, HIIE consisting of four 4 min cycling bouts at 90–95% of HRmax, separated by 3 min active recovery at 70% HRmax, increased both nuclear and mitochondrial DNA damage and was accompanied by increased lipid hydroperoxides and ascorbyl free-radical formation, indicating a substantial exercise-induced redox perturbation. Acute MitoQ administration before exercise did not prevent these responses, whereas 21 days of MitoQ supplementation attenuated exercise-induced nuclear and mitochondrial DNA damage in lymphocytes and mitochondrial DNA damage in skeletal muscle (doi: 10.1016/j.redox.2020.101673). mtROS do not appear to be the sole upstream trigger for PGC-1α. In 23 untrained middle-aged men, HIIE (10 × 60 s at peak power) increased skeletal-muscle PPARGC1A mRNA after 3 h, with a greater response following MitoQ compared to placebo, despite there being no significant changes in mtDNA damage, 4-HNE, or systemic oxidative-stress markers. These findings support a multifactorial regulation of exercise-induced PGC-1α signaling rather than an obligatory mtROS-dependent mechanism [44].

Ca^2+^ signaling is another mechanism by which excitation–contraction coupling during exercise translates into mitochondrial adaptation. Each excitation–contraction cycle leads to an increase in cytoplasmic calcium, which activates the contractile machinery and engages Ca^2+^/calmodulin-dependent signaling pathways, in which CaMKII plays a key role. In mice with CaMKIIγ/δ-deficient muscle, no improvement in exercise capacity was observed after four weeks of training, primarily due to pronounced reductions in OXPHOS and glycolysis, as well as an increase in mitochondrial ultrastructural abnormalities [45].

CaMKII serves as a convergence point between Ca^2+^/calmodulin-dependent signaling and redox signaling, as the oxidation of regulatory Met281/282 residues can sustain CaMKII activity. Oxidation-resistant CaMKII, generated by replacing methionine with valine, exhibited attenuated contraction and reduced intracellular Ca^2+^ levels, which blunted the acute skeletal muscle transcriptional response to exercise [46].

Another node where various metabolic pathways converge is AMPK, which is activated during exercise by ADP, AMP, and inorganic phosphate, signaling events initiated by PCr. A mathematical model of skeletal-muscle bioenergetics and AMPK signaling, calibrated against human exercise data, demonstrated that during submaximal exercise, PCr levels drop rapidly, accompanied by a rise in ADP and AMP that activates AMPK. The same model indicates that, although AMP is a much more potent activator of AMPK, the enzyme’s activity must be attributed to the broader adenine–nucleotide environment generated by exercise [39].

AMPK activation can promote PGC-1α-dependent signaling through mechanisms involving SIRT1, an NAD^+^-dependent deacetylase that regulates PGC-1α activity through deacetylation.

Repeated training modifies the magnitude of the acute signaling response to a fixed absolute workload. Before training, a single HIIE session increased nuclear PGC-1α protein 3.1-fold and PGC-1α mRNA 3.6-fold after 3 h. However, after 40 HIIT sessions performed twice daily for 20 consecutive days, the same absolute exercise no longer significantly increased nuclear PGC-1α protein or PGC-1α mRNA. Training increased lactate-threshold power by 9% and VO_2_peak by 11.7%, making the same workload a lower relative metabolic challenge. These findings suggest that mitochondrial adaptation progressively attenuates the acute molecular signaling required to stimulate further mitochondrial biogenesis when the same exercise stimulus is repeated [47].

**Figure 4:**
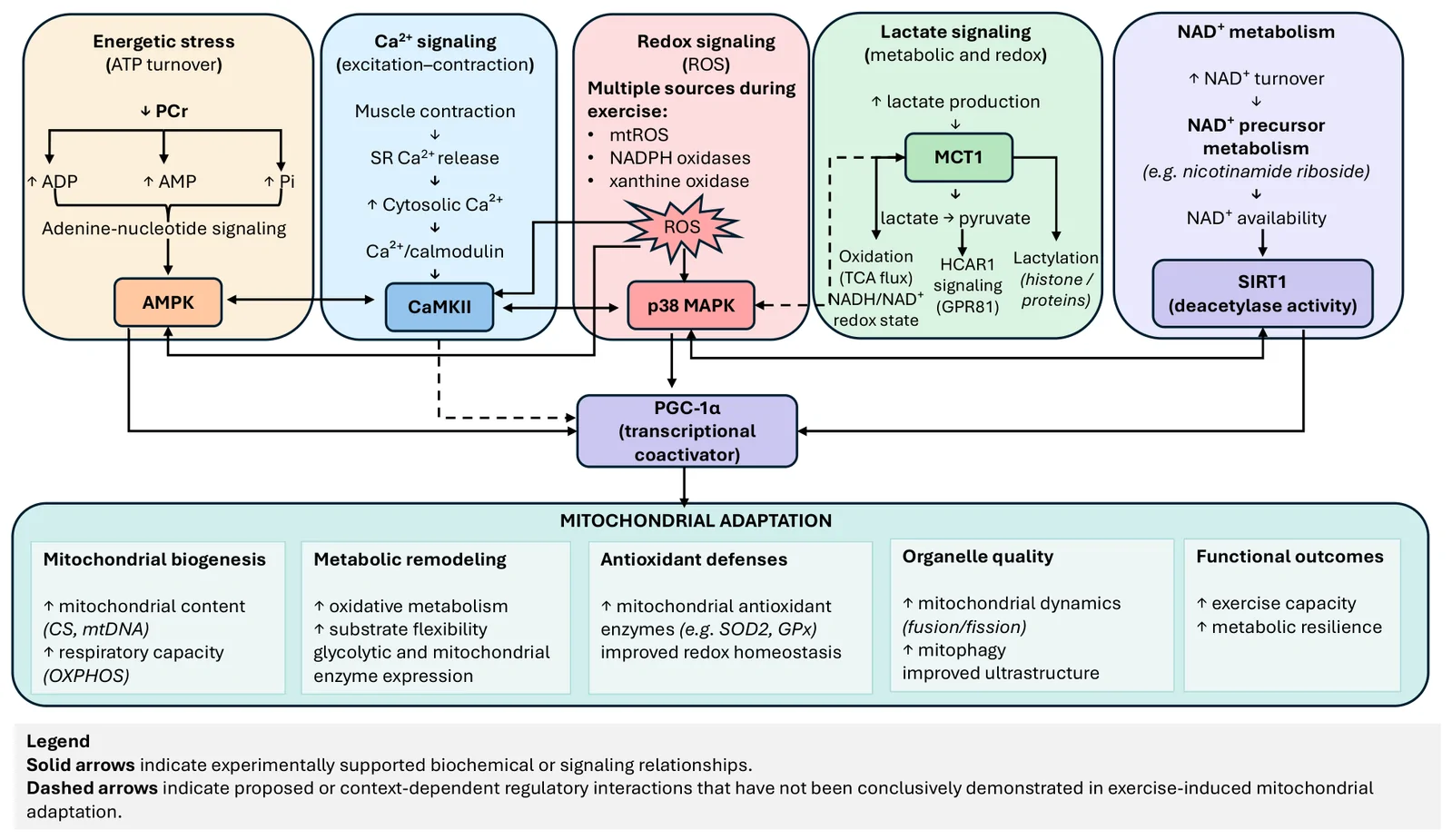
Integrated signaling network linking exercise-induced metabolic stress to mitochondrial adaptation. Abbreviations: ADP—adenosine diphosphate; AMP—adenosine monophosphate; AMPK—AMP-activated protein kinase; CaMKII—Ca^2+^/calmodulin-dependent protein kinase II; CS—citrate synthase; GPx—glutathione peroxidase; HCAR1—hydroxycarboxylic acid receptor 1; MCT1—monocarboxylate transporter 1; mtDNA—mitochondrial DNA; mtROS—mitochondrial reactive oxygen species; NAD^+^—nicotinamide adenine dinucleotide, oxidized form; NADH—nicotinamide adenine dinucleotide, reduced form; OXPHOS—oxidative phosphorylation; PCr—phosphocreatine; PGC-1α—peroxisome proliferator-activated receptor gamma coactivator 1-alpha; Pi—inorganic phosphate; ROS—reactive oxygen species; SIRT1—sirtuin 1; SOD2—superoxide dismutase 2; SR—sarcoplasmic reticulum, ↓—down arrow means decreased effect, ↑—up arrow means increased effect.

Recent experimental data indicate that the AMPK → SIRT1 → PGC-1α relationship is not a simple linear cascade of activation. Administration of nicotinamide riboside to young, healthy men altered several components of the skeletal muscle NAD^+^ metabolome without increasing muscle NAD^+^ content and did not enhance exercise-induced SIRT1 or SIRT3 signaling or the post-exercise increase in PGC-1α mRNA. This demonstrates a complex, multifactorial relationship and shows that manipulating NAD^+^ precursor metabolism is insufficient to potentiate SIRT1–PGC-1α signaling during acute exercise (see Figure 4) [48].

Importantly, lactate may intersect with this adaptive network at multiple levels, including MCT1-dependent transport and oxidation, NAD^+^–SIRT1–PGC-1α signaling, redox-sensitive pathways, HCAR1-mediated signaling, and lactylation, thereby providing potential links between metabolic stress and mitochondrial remodeling that will be discussed in detail in the following sections.

### 4.3. Mitohormesis: When Mitochondrial Stress Becomes Beneficial

Mitohormesis occurs when transient mitochondrial stress acts as an adaptive rather than a destructive pathway. It is triggered by a combination of rapid ATP consumption, Ca^2+^ flux, and oxidative stress. If the duration and intensity of these processes become significant, compensatory mechanisms are activated to restore mitochondrial homeostasis and gradually increase resistance to metabolic challenges. However, persistent mitochondrial stress that exceeds cellular repair capacity contributes to mitochondrial dysfunction.

ROS are important components of mitohormetic signaling. In 23 untrained middle-aged men, 10 days of MitoQ supplementation augmented the skeletal-muscle PGC-1α mRNA response 3 h after acute HIIT, despite there being no detectable changes in systemic or muscle oxidative-stress markers. However, MitoQ did not enhance subsequent training-induced mitochondrial content or respiration, suggesting modulation of acute mitochondrial adaptive signaling rather than mitochondrial remodeling itself [44].

A single bout of aerobic exercise in mice activated c-Jun N-terminal kinase (JNK) signaling and increased skeletal-muscle UPR^mt-associated genes and proteins, including HSP60, ClpP, Lonp1, Yme1L1, and ATF5. Pharmacological JNK inhibition with SP60012 attenuated the induction of UPR^mt markers and removed exercise-induced increases in several OXPHOS proteins. Sustained effort involving four weeks of aerobic training, on the other hand, increased OXPHOS protein abundance, mitochondrial respiratory capacity, and aerobic performance, consistent with an association between repeated transient UPR^mt activation and mitochondrial adaptation [49].

Mitochondrial quality-control mechanisms are activated during exercise. A single bout of unilateral resistance exercise rapidly altered skeletal-muscle mitochondrial dynamics, increasing DRP1^Ser616 phosphorylation within 1 h and producing ultrastructural abnormalities of mitochondrial cristae together with a shift toward a mitochondrial fission phenotype. Despite reduced mitochondrial localization of the mitophagy-related proteins PARKIN and BNIP3L/NIX, electron microscopy revealed mitophagosome-like structures and mitochondria undergoing extrusion from the muscle fiber, suggesting that acute resistance exercise activates multiple, potentially complementary mitochondrial quality-control mechanisms [43].

Therefore, the distinction between beneficial mitohormesis and maladaptive mitochondrial stress seems to depend on the magnitude, duration, and reversibility of the challenge relative to cellular repair capacity.

Importantly, the experimental context must be considered when interpreting mitohormetic responses. Genetic models involving sustained alterations in mitochondrial function provide valuable insights into stress-responsive signaling and compensatory mechanisms, but they do not necessarily reproduce the transient and reversible mitochondrial perturbations associated with physical exercise. In contrast, repeated exercise-induced metabolic challenges can promote mitochondrial remodeling and improved oxidative capacity without requiring persistent mitochondrial dysfunction. Therefore, the findings from genetically induced mitochondrial stress should be interpreted as mechanistic evidence of cellular adaptive potential rather than direct confirmation of equivalent responses during exercise.

### 4.4. Myokines, Exerkines, and Mitokines

Mitochondria can communicate with distant cells and organs via mitokines. The terms “mitokines,” “myokines,” and “exerkines” describe overlapping forms of inter-organ communication during physical exercise. Myokines are released by skeletal muscle, while exerkines are released from various tissues during exercise; mitokines, meanwhile, are produced in response to mitochondrial stress, regardless of their tissue of origin. A molecule released from skeletal muscle in response to exercise-induced mitochondrial stress may simultaneously function as a myokine, an exerkine, and a mitokine.

Mitokines include nuclear-encoded stress-responsive proteins, such as FGF21 and GDF15, as well as mitochondrial DNA-encoded peptides, such as MOTS-c and Humanin. These factors differ in their genetic origin but can participate in cellular and interorgan communication in response to mitochondrial stress (Table 1).

The classification of FGF21 and GDF15 as mitokines is therefore based on their stress-responsive functions rather than their genetic origin. Both factors can be induced under specific conditions of mitochondrial dysfunction or stress and contribute to systemic metabolic signaling, although their expression is not exclusively regulated by mitochondrial stress. For example, muscle-specific mitochondrial stress in mice induced GDF15 as a myokine, while genetic deletion of Gdf15 abolished systemic modifications [50].

**Table 1:** Major exercise-responsive mitokines and mitochondrial-derived signals.

Molecular Origin/Source	Exercise Model	Mitochondrial-Stress Link	Exercise Response	Proposed Mechanism	Major Target/Effect	Human Evidence	Classification	Ref.
FGF21								
Nuclear-encoded protein (FGF21 gene); predominantly liver-derived, with skeletal muscle expression under mitochondrial stress	Acute endurance exercise/exercise training/muscle mitochondrial stress	MD, ISR, ATF4- stress signaling	↑ Circulating FGF21 in acute/prolonged exercise; (exercise and metabolic context response)	FGFR1c/β-Klotho and metabolic remodeling	Adipose tissue, liver, CNS; glucose/lipid metabolism, thermogenesis and metabolic homeostasis	Yes	Stress-responsive mitokine/hepatokine/context-dependent exerkine	[51,52]
GDF15								
Nuclear-encoded protein (GDF15 gene); liver/hepatosplanchnic tissues and other stress-responsive tissues, including skeletal muscle	Resistance, endurance exercise, prolonged/exhaustive exercise	Muscle mitochondrial respiratory uncoupling	↑ after resistance and endurance exercise; (↑↑ prolonged exercise)	GFRAL/RET signaling	CNS, adipose (WAT browning), energy balance, insulin sensitivity	Yes	Stress-responsive mitokine/context-dependent exerkine	[50,53,54,55]
MOTS-c								
mtDNA-derived peptide; encoded by a short open reading frame within the mitochondrial 12S rRNA region (MT-RNR1)	Acute cycling/HIIT; endurance and resistance exercise	Metabolic stress induces nuclear translocation; integrates with AMPK–PGC-1α and stress-responsive transcription	Human muscle MOTS-c ↑ ~11.9-fold after acute exercise; circulating MOTS-c ↑ ~1.5–1.6-fold; responses vary by protocol	AMPK signaling; stress-dependent nuclear translocation; HSF1-stress adaptation	Skeletal muscle; metabolic flexibility, glucose handling	Yes, but limited and protocol-dependent	MDP/potential mitokine	[5,56,57,58]
Humanin								
mtDNA-derived peptide; encoded by a short open reading frame within the mitochondrial 16S rRNA region (MT-RNR2)	Acute HIIT; short-term HIIT; endurance/resistance	Cellular-stress-responsive MDP	Acute HIIT ↑ skeletal-muscle and plasma Humanin; isolated muscle contraction also ↑ Humanin	Cytoprotective and metabolo-protective signaling; mechanisms of exercise response incompletely resolved	Skeletal muscle and multiple metabolic tissues	Yes	MDP/exercise-responsive mitochondrial signal; potential exerkine	[59]

Abbreviations: ATF4—activating transcription factor 4; CNS—central nervous system; FGF21—fibroblast growth factor 21; FGFR1c—fibroblast growth factor receptor 1c; ISR—integrated stress response; MD—mitochondrial dysfunction; GDF15—growth differentiation factor 15; GFRAL—GDNF family receptor alpha-like; RET—rearranged during transfection receptor tyrosine kinase; WAT—white adipose tissue; AMPK—AMP-activated protein kinase; HIIT—high-intensity interval training; HSF1—heat shock factor 1; MDP—mitochondrial-derived peptide; MOTS-c—mitochondrial open reading frame of the 12S rRNA type-c; mtDNA—mitochondrial DNA; PGC-1α—peroxisome proliferator-activated receptor gamma coactivator 1-alpha; ↑—up arrow means increased effect.

#### 4.4.1. FGF21: Linking Mitochondrial Stress to Systemic Metabolic Adaptation

FGF21 is a nuclear-encoded endocrine factor produced predominantly by the liver, which represents a major contributor to circulating FGF21 concentrations. Skeletal muscle can also express and produce FGF21 under specific conditions of mitochondrial stress or dysfunction [60,61]. However, increased circulating FGF21 concentrations following exercise should not be interpreted as direct evidence of skeletal muscle secretion, since the relative contributions of hepatic and muscular sources cannot be established from circulating measurements alone.

Importantly, studies assessing circulating FGF21 concentrations alone cannot establish its tissue of origin, whereas tissue-specific expression analyses and measurements of organ-specific secretion provide more direct evidence of hepatic or muscular contributions.

Mitochondrial stress activates the integrated stress response (ISR), in which eIF2α-dependent signaling promotes ATF4 activity and transcription of stress-responsive genes, including FGF21. The mitochondrial stress–ISR–ATF4–FGF21 axis provides a mechanism that converts intracellular mitochondrial disturbance into an endocrine signal.

Evidence for the functional involvement of FGF21 in mitochondrial stress adaptation comes primarily from genetically modified mouse models. In double knock-out mice, hepatic deletion of Crif1 and Fgf21 led to OXPHOS/ISR^mt dysfunction. Loss of FGF21 impaired glucose clearance and attenuated insulin signaling, thermogenic gene expression, and energy expenditure in white adipose tissue, showing that stress-induced FGF21 mediates cell-non-autonomous metabolic adaptation to mitochondrial dysfunction [51]. Similarly, in UCP1-transgenic/Fgf21-knockout mice (TG/Fgf21−/− mice), elimination of FGF21 reduced mitochondrial stress-induced browning of white adipose tissue, whereas obesity resistance, improved glycemic control, hepatic lipid protection, and the cell-autonomous muscle mitohormetic response remained largely preserved in its absence, indicating that FGF21 mediates a specific arm of the systemic mitochondrial stress response [52]. These studies indicate that FGF21 mediates selected systemic consequences of mitochondrial stress rather than being universally required for all mitohormetic adaptations.

Although exercise-associated metabolic and mitochondrial stress may engage overlapping signaling mechanisms, the contribution of skeletal muscle-derived FGF21 to exercise adaptation remains incompletely established. Importantly, the studies described above demonstrate mitochondrial stress-dependent FGF21 signaling but do not establish lactate as its upstream regulator.

During exercise, the mitochondrial stress–ATF4–FGF21 pathway could represent a plausible mechanism explaining the influence of metabolic stress on inter-organ communication. However, the adaptive changes induced by exercise-related mitochondrial stress should not be equated with experimental models of mitochondrial dysfunction, and the role of skeletal muscle-derived FGF21 as an exercise-induced mitokine remains to be established.

Thus, mitochondrial stress–FGF21 signaling represents a well-supported component of systemic metabolic adaptation, but its direct coupling to exercise-induced lactate signaling remains a separate mechanistic question. Evidence addressing lactate-dependent FGF21 regulation, including experimental manipulation of lactate availability and receptor signaling, is critically examined in Section 4.1.

#### 4.4.2. GDF15: From Mitochondrial Stress to Systemic Energy Balance

GDF15 is a nuclear-encoded, stress-responsive endocrine factor expressed in multiple tissues, including the liver, heart, and skeletal muscle. Although mitochondrial dysfunction can induce GDF15 expression and contribute to systemic metabolic signaling, its production is not restricted to mitochondrial stress. Circulating GDF15 concentrations may also increase in response to broader metabolic and cellular disturbances. Therefore, exercise-induced elevations in circulating GDF15 should be interpreted as systemic stress responses rather than direct evidence of mitochondrial stress or secretion from contracting skeletal muscle.

Using UCP1-transgenic mice with mitochondrial respiratory uncoupling crossed with whole-body Gdf15-knockout mice, it was demonstrated that genetic loss of Gdf15 abolished stress-induced white adipose tissue browning, and enhanced systemic insulin sensitivity and metabolic flexibility without altering the local muscle mitochondrial stress response or muscle wasting. An important characteristic of GDF15 is the ability to couple cellular stress to appetite. Gdf15-competent mice presented with daytime anorexia, which was not manifested in those with deletion of Gdf15. Interestingly, the anorectic action was largely absent during the nocturnal feeding period, revealing that the metabolic effects of endogenous GDF15 depend on physiological context and circadian timing [50]. Although these findings demonstrate exercise-induced Gdf15 expression in multiple tissues, they do not establish the relative contribution of each tissue to circulating GDF15 concentrations.

The secretion of GDF15 is responsive to both resistance and endurance exercise. In mice subjected to exhaustive treadmill running, both GDF15 and *Gdf15* in liver, heart, and skeletal muscle increased. However, both wild-type and GFRAL-knockout mice showed similar reductions in voluntary running and food intake after exhaustive exercise, indicating that these acute post-exercise responses were not dependent on endogenous GDF15–GFRAL signaling. In contrast, exogenous administration of recombinant GDF15 reduced voluntary running in wild-type mice, but not in GFRAL-knockout animals [53]. Although GDF15 is capable of modulating exercise-related behavior through GFRAL-dependent mechanisms, its endogenous increase after strenuous exercise is not necessarily responsible for the acute behavioral and metabolic responses observed.

Whilst Ost et al. (2020) showed that GDF15 mediates specific systemic metabolic adaptations to sustained muscle mitochondrial dysfunction, the study by Klein et al. (2021) showed that the characteristic increase in GDF15 following acute strenuous exercise is not required for the immediate post-exercise suppression of food intake or voluntary activity, highlighting the context-dependent actions of GDF15 [50,53].

Human studies further indicate that exercise-induced GDF15 secretion is strongly influenced by the systemic endocrine–metabolic environment Sampling of arterial and hepatic venous blood in healthy men revealed significant hepatosplanchnic GDF15 release during exercise. Experimentally increasing the glucagon/insulin ratio at rest resulted in a threefold increase in circulating GDF15, whereas preventing the normal exercise-induced change in this ratio via a pancreatic clamp abolished the exercise-induced GDF15 response. Consequently, GDF15 release is linked to the endocrine–metabolic environment generated by exercise and not directly to skeletal muscle contraction [54]. These findings provide experimental evidence that hormonal regulation contributes to exercise-induced GDF15 secretion and challenge the assumption that contracting skeletal muscle is its principal source.

GDF15 is considered GDF15 an acute exercise-responsive stress signal. In overweight or obese physically inactive adults, a single bout of moderate-intensity cycling increased circulating GDF15 by 18% immediately after exercise and by 32% after 60 min of recovery. In case of chronic effort, six months of either moderate- or vigorous-intensity exercise did not change fasting GDF15 concentrations or the GDF15 response to an acute exercise bout [55].

GDF15 is a context-dependent mitokine/exerkine acting both as a mitochondrial stress signal and as an exercise-responsive factor. Severe or sustained mitochondrial stress can generate a GDF15-dependent endocrine response that modifies appetite, adipose-tissue remodeling, insulin sensitivity, and systemic energy metabolism, whereas the transient physiological increase in GDF15 produced by exercise does not necessarily reproduce these effects. Although lactate accumulation and GDF15 secretion may occur under increased metabolic demand, their concurrent responses do not establish direct regulatory coupling. Moreover, the experimentally demonstrated contribution of the glucagon-to-insulin ratio provides an alternative mechanism for exercise-induced GDF15 release. Therefore, whether physiological changes in lactate availability directly influence GDF15 expression or secretion during exercise remains unresolved and requires targeted experimental investigation.

#### 4.4.3. MOTS-c: A Mitochondrial-Derived Peptide Linking Metabolic Stress to Exercise Adaptation

MOTS-c is a mitochondrial DNA-encoded peptide involved in the regulation of glucose and lipid metabolism, cellular stress resistance, and metabolic homeostasis, with several of its biological effects associated with AMPK signaling. Unlike nuclear-encoded stress-responsive factors such as FGF21 and GDF15, MOTS-c originates from the mitochondrial genome and may contribute to communication between mitochondrial metabolic status and nuclear adaptive responses. MOTS-c can translocate to the nucleus under metabolic stress and regulate nuclear gene expression. In C2C12 myoblasts exposed to glucose restriction and serum deprivation, fluorescently labeled exogenous MOTS-c showed time-dependent nuclear translocation. At the same time, MOTS-c is responsive to exercise in humans. In sedentary healthy young men, acute cycling increased endogenous skeletal-muscle MOTS-c approximately 11.9-fold. Animal experiments showed that exogenous MOTS-c enhanced physical performance across young, middle-aged, and old mice; notably, two weeks of treatment in old mice increased running time approximately twofold and running distance 2.16-fold. MOTS-c modified glycolysis and amino-acid metabolism, whereas comparable changes were not detected in non-exercised muscle [5]. Although these findings support the involvement of MOTS-c in metabolic stress responses and exercise-related adaptation, they do not establish whether exercise-induced lactate production directly regulates MOTS-c expression or nuclear translocation.

The exercise-responsiveness of MOTS-c appears to vary according to the experimental protocol and the biological compartment investigated. In a randomized study of 30 recreationally active adults allocated to endurance exercise, resistance exercise, or a control, 45 min of cycling at 70% of estimated VO_2_max increased circulating Humanin and produced a trend toward increased circulating MOTS-c, although the latter response showed considerable interindividual variability. In contrast, skeletal-muscle MOTS-c remained unchanged following either exercise modality, with endurance exercise increasing PGC-1α-related transcripts and resistance exercise inducing c-Myc and 45S pre-rRNA [56]. These findings contrast with the pronounced skeletal-muscle MOTS-c response reported in the earlier acute cycling study suggesting that exercise-induced changes in MOTS-c are not uniform across populations, protocols, or sampling compartments.

The relationship between MOTS-c and AMPK–PGC-1α signaling is complex and bidirectional. Thus, in high-fat-diet (HFD) obese mice, skeletal-muscle and circulating MOTS-c levels were reduced, whereas eight weeks of treadmill training (60 min/day, 5 days/week) increased muscle and plasma MOTS-c, together with PGC-1α and GLUT4, and enhanced phosphorylation of AMPK. AMPK inhibition with Compound C in C2C12 myotubes reduced both PGC-1α and MOTS-c expression, while AMPK activation with AICAR increased their expression. Exogenous MOTS-c increased AMPK and acetyl-CoA carboxylase (ACC) phosphorylation and PGC-1α expression, supporting a feed-forward interaction between MOTS-c and the AMPK–PGC-1α axis [57]. These results support reciprocal interactions between MOTS-c and AMPK-associated metabolic signaling. However, AMPK is activated by multiple exercise-related stimuli, including changes in cellular energy status, and even indirectly in lactate by modifying NAD^+^/NADH ratio.

Nevertheless, the interpretation of circulating MOTS-c responses is constrained by several methodological limitations. The relatively small sample sizes of available human studies may limit statistical power, particularly in the presence of substantial interindividual variability. Furthermore, differences in analytical methods and the limited standardization of circulating MOTS-c measurements may complicate comparisons across studies. Variations in exercise modality, intensity, duration, and blood sampling time may also contribute to inconsistent findings. Consequently, circulating MOTS-c responses should be interpreted cautiously until larger studies using validated analytical approaches and standardized exercise protocols become available.

In addition to its influence on glucose metabolism, MOTS-c may contribute to the preservation of skeletal muscle homeostasis under conditions of immobilization-induced stress. Administration of MOTS-c (15 mg/kg/day) to male C57BL/6J mice subjected to hindlimb immobilization for 8 days attenuated the loss of skeletal muscle mass by limiting lipid infiltration during disuse and restored AKT–FOXO1/FOXO3a signaling [58].

Although experimental studies involving exogenous MOTS-c administration support its functional effects on metabolic signaling and physical performance, changes in endogenous MOTS-c concentrations following exercise primarily provide associative evidence. These findings do not establish that exercise-induced MOTS-c is directly responsible for skeletal muscle mitochondrial adaptation, and its causal contribution to human training responses remains to be demonstrated.

The proposed lactate–MOTS-c interaction is currently supported primarily by convergence to metabolic signaling pathways rather than direct experimental evidence. Whether exercise-induced lactate availability influences endogenous MOTS-c expression, nuclear translocation, or circulating concentrations remains unresolved. Establishing such a relationship will require controlled manipulation of lactate availability or transport, combined with simultaneous assessment of MOTS-c responses and AMPK signaling during exercise.

Similar limitations apply to Humanin, another mitochondrial-derived peptide whose circulating concentrations have been reported to increase following acute endurance exercise [56]. However, these findings primarily demonstrate an exercise-associated endocrine response and do not establish a direct functional contribution of endogenous Humanin to skeletal muscle mitochondrial adaptation. Its tissue-specific regulation, the influence of different exercise protocols, and its potential causal involvement in training-induced mitochondrial remodeling remain insufficiently characterized.

## 5. The Lactate–Mitokine Axis: Convergence of Glycolytic and Mitochondrial Stress Signaling

### 5.1. From Parallel Signals to an Integrated Lactate–Mitokine Network

During physical exertion, a simultaneous increase in the rate of glycolysis, accompanied by lactate production and mitochondrial activation, occurs, leading to endocrine changes. The proposed lactate–mitokine axis integrates findings of different mechanistic strengths, ranging from exercise-associated changes in circulating factors to experimentally demonstrated, tissue-specific regulatory mechanisms. These findings should therefore be interpreted according to their experimental context and causal relevance, as critically summarized in Table 2.

In young men exposed to moderate-, vigorous-, and high-intensity interval cycling, HIIE produced the highest amount of lactate and circulating FGF21; these exercise-induced changes in lactate were correlated positively with changes in FGF21, supporting an association between glycolytic stress and FGF21 release without establishing a causal relationship [62].

Intracerebroventricular administration of lactate to rats increased hypothalamic FGF21 mRNA and cerebrospinal fluid (CSF) FGF21 levels, whereas pharmacological inhibition of GPR81 or p38-MAPK abolished this response. In male Wistar rats, acute endurance exercise increased cerebrospinal fluid FGF21 concentrations by approximately 95% and selectively increased FGF21 mRNA and protein expression in the hypothalamus. Activation of p38-MAPK and ERK1/2 signaling increased `circulating catecholamines and enhanced adipose-tissue cAMP signaling and HSL phosphorylation. Blocking brain lactate transport with α-cyano-4-hydroxycinnamate before a 60 min bout of endurance exercise attenuated exercise-induced hypothalamic FGF21 expression, sympathetic activation, and fat mobilization. Moreover, intracerebroventricular lactate administration at rest reproduced an increase in hypothalamic FGF21, whereas pharmacological inhibition of either GPR81 or p38-MAPK abolished the lactate-induced FGF21 response. These findings provide experimental support for a lactate-dependent hypothalamic FGF21 response involving GPR81 and p38-MAPK signaling in rats. Although these findings provide mechanistic evidence for lactate-dependent hypothalamic FGF21 regulation in rats, several limitations should be considered. Intracerebroventricular lactate administration bypasses physiological lactate transport and distribution, while pharmacological interventions targeting central lactate signaling do not fully reproduce the complex metabolic and neuroendocrine conditions of exercise. Moreover, hypothalamic FGF21 expression and cerebrospinal fluid concentrations cannot be directly equated with peripheral FGF21 secretion, which is predominantly hepatic under physiological conditions. Therefore, these findings support a tissue-specific central regulatory mechanism but do not establish that exercise-derived lactate directly controls circulating FGF21 concentrations in humans [63].

### 5.2. Lactate Availability and Mitochondrial Metabolic Remodeling

Experimental manipulation of lactate availability indicates that lactate exposure can directly remodel mitochondrial function.

In male ICR mice, four weeks of sodium lactate administration at a dose that transiently raised blood lactate levels to approximately 20 mM selectively increased Complex I-driven state 3 mitochondrial respiration supported by pyruvate plus malate or glutamate plus malate in skeletal muscle; this was accompanied by increased Complex I + III and IV activities, with the Complex I subunit NDUFS4 showing a particularly significant increase. In contrast, Complex II-dependent respiration (with succinate plus rotenone) decreased, while fatty acid-fueled respiration remained unchanged [67].

Lactate administration can remodel skeletal muscle oxidative metabolism in the absence of exercise. In C57BL/6J mice fed an HFD, lactate administered five days a week for eight weeks increased palmitate oxidation by enhancing the activity of β-oxidation enzymes (long-chain acyl-CoA dehydrogenase [LCAD] and short-chain acyl-CoA dehydrogenase [SCAD]), citrate synthase, and pyruvate carboxylase, as well as levels of malate and aspartate, findings consistent with the malate–aspartate shuttle [64].

Data from the literature indicates three possible interconnected mechanisms by which lactate influences mitochondrial function: the oxidation of lactate to pyruvate fuels and increases the TCA cycle rate; the lactate/pyruvate conversion alters the NADH/NAD^+^ redox environment; and MCT-dependent control of intracellular lactate availability influences mitochondrial remodeling. However, changes in mitochondrial oxidative capacity, respiratory chain activity, or substrate utilization reflect metabolic remodeling and do not necessarily indicate activation of mitochondrial stress-responsive signaling. In particular, the available lactate administration studies do not establish whether these metabolic adaptations involve activation of the mitochondrial integrated stress response (mtISR), including ATF4-associated pathways implicated in FGF21 and GDF15 induction. Therefore, although lactate availability can modulate mitochondrial function, its capacity to trigger stress-responsive mitokine production remains to be demonstrated.

Establishing a causal relationship between lactate signaling and mitochondrial stress-responsive mitokine production will require experimental approaches that assess each step of the proposed pathway. In vitro studies could combine controlled lactate exposure with genetic or pharmacological manipulation of lactate transport (MCT1/MCT4) and HCAR1 signaling, while monitoring mitochondrial respiration, redox balance, and activation of stress-responsive pathways, particularly the integrated stress response involving eIF2α–ATF4 signaling. These measurements should be accompanied by quantification of FGF21 and GDF15 expression and secretion to determine whether lactate-induced metabolic changes translate into endocrine responses. Inhibition or genetic disruption of candidate stress-signaling pathways would help establish whether mitokine induction depends on these mechanisms. Exercise studies incorporating lactate manipulation, tissue-specific measurements, and circulating mitokine profiling could then assess the physiological relevance of these interactions.

### 5.3. Shared Molecular Nodes Connecting Lactate and Mitokine Signaling

**Figure 5:**
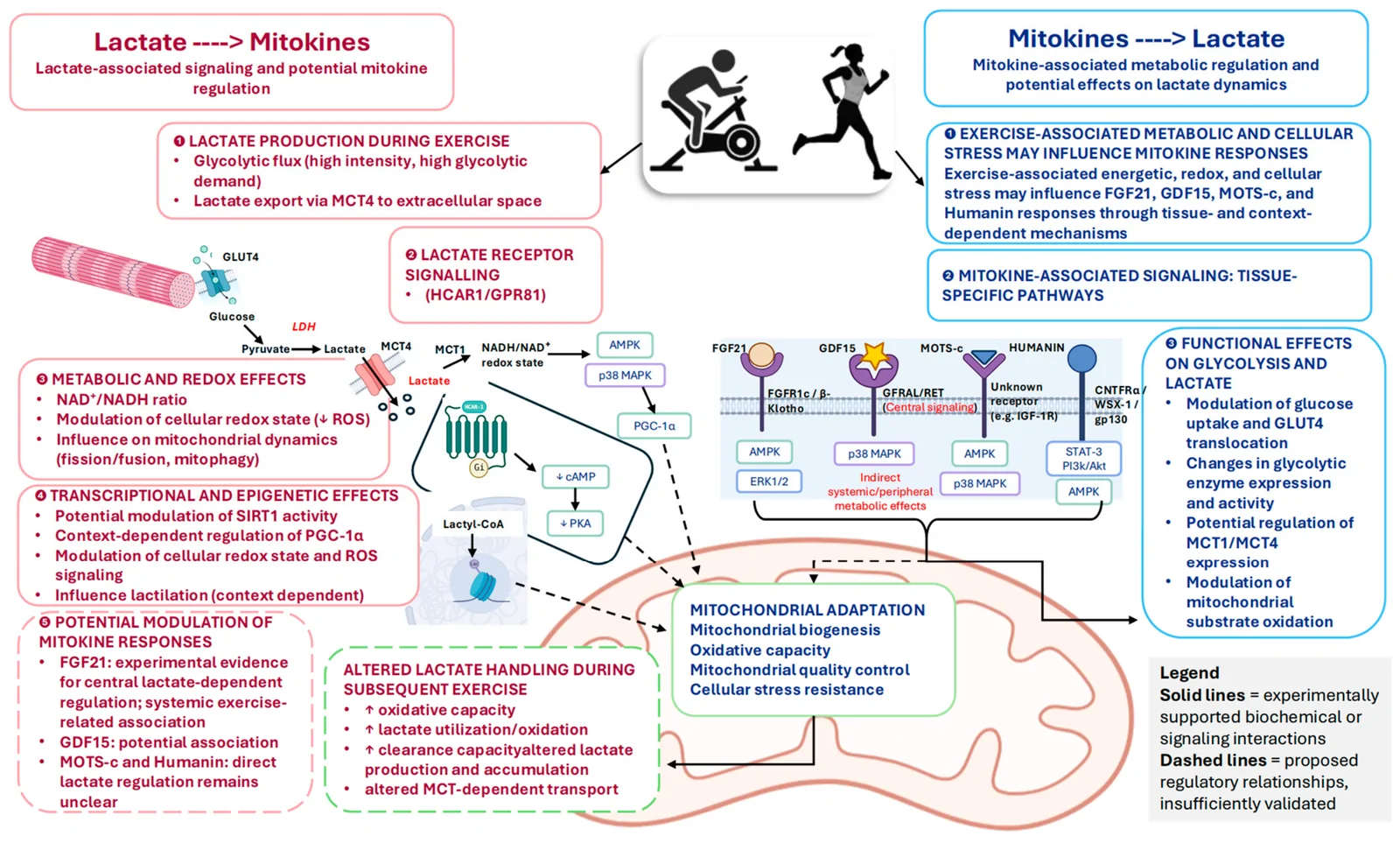
Proposed bidirectional interactions between lactate metabolism and mitokine signaling Note: The proposed bidirectional lactate–mitokine axis should therefore be regarded as a conceptual framework rather than a fully established causal signaling pathway. ↓—down arrow means decreased effect; ↑—up arrow means increased effect; solid arrows and lines indicate experimentally supported biochemical or signaling relationships, dashed arrows and lines indicate proposed or context-dependent regulatory interactions that have not been conclusively demonstratedCreated in BioRender. Tero-Vescan, A. (2026) https://BioRender.com/q7zwzdd (accessed on 1 October 2026).

AMPK represents one of the strongest candidate convergence points. In mice with skeletal muscle PGC-1α overexpression, lactate production was approximately twice as high as in wild-type mice at the same relative exercise intensity, simulated for a blood lactate concentration of 4 mM. PGC-1α overexpression also increased lactate uptake/clearance capacity, an effect associated with elevated skeletal muscle MCT1 levels, citrate synthase activity, and oxidative capacity. In the same study, C57BL/6J mice performing treadmill exercise at progressively increasing intensities exhibited, at the highest intensity tested, a concomitant rise in calculated lactate production and the p-AMPK/total AMPK ratio in skeletal muscle. It is important to mention that this association does not prove lactate is the direct cause of AMPK activation, as both responses may reflect the more pronounced energetic and metabolic stress induced by high-intensity exercise [65]. For more details, see Figure 5.

However, AMPK activation and PGC-1α-associated transcriptional responses are not specific to lactate signaling, as they can also be influenced by energetic stress, muscle contraction, calcium-dependent signaling, and changes in cellular redox status. Accordingly, their involvement in both lactate-associated metabolic responses and mitokine-mediated signaling should be interpreted as evidence of molecular convergence rather than direct communication between these systems. Although experimental interventions support specific relationships, such as the involvement of AMPK in MOTS-c-mediated mitochondrial effects, a direct lactate-dependent regulation of mitokine production through these shared pathways remains a mechanistic hypothesis.

Repeated MOTS-c administration in skeletal-muscle-specific AMPKα1/α2 knockout mice showed that several MOTS-c-induced increases in mitochondrial OXPHOS capacity and the reduction in H_2_O_2_ emissions observed in control muscle were blunted or absent in AMPK-deficient muscle, indicating that AMPK is partially required for the mitochondrial bioenergetic effects of MOTS-c [66].

Although these studies identify AMPK as a molecular node involved in both exercise-induced metabolic stress responses and MOTS-c-mediated mitochondrial regulation, they do not demonstrate a direct lactate–AMPK–MOTS-c signaling pathway. Exercise-induced AMPK activation may occur independently of lactate accumulation, primarily in response to cellular energetic stress. Similarly, the AMPK-dependent effects of MOTS-c do not establish that lactate controls endogenous MOTS-c production. Convergence at AMPK should be interpreted as a mechanistic hypothesis for potential pathway interaction rather than evidence of direct regulatory coupling. A second point of convergence is the NADH/NAD^+^ redox couple; since the reaction catalyzed by LDH is reversible, changes in lactate flux can transfer reducing equivalents into the mitochondria. Although studies do not establish a direct lactate–SIRT1–mitokine pathway, they do identify the NADH/NAD^+^ ratio as a plausible biochemical interface between glycolytic and mitochondrial signaling.

Another in vivo study using MCT1 knockout mice and in vitro pharmacological MCT1 inhibition with AZD3965 in C2C12 myotubes demonstrated that altering lactate transport increased the NAD^+^/NADH ratio and SIRT1 activity, while reducing PGC-1α acetylation. Blocking the MCT1 lactate transporter shifted skeletal muscle substrate utilization toward glucose, increasing glycolytic and glucose-derived TCA cycle flux while reducing the contribution of circulating lactate to mitochondrial metabolism [68].

Redox signaling provides a third, albeit more complex, interface. The increase in mitochondrial energy metabolism during physical exertion enhances electron transport and ROS production. Lactate increased intracellular ROS in C2C12 myotubes in a dose-dependent manner, and ROS scavenging with N-acetylcysteine attenuated lactate-induced ERK1/2 and p38-MAPK activation, supporting the ROS-dependent engagement of redox-sensitive signaling pathways [69].

Finally, PGC-1α may represent a downstream convergence and feedback node rather than a component of a dedicated lactate pathway. A single bout of high-intensity exercise increased hippocampal PGC-1α mRNA and mtDNA copy number in mice, whereas the pharmacological inhibition of MCT with UK5099 attenuated both responses without altering exercise performance or circulating lactate levels. These results suggest the role for exercise-derived lactate uptake in PGC-1α-associated mitochondrial biogenesis [70].

Lactate can influence mitochondrial remodeling, but the lactate–mitochondria axis can also operate in the reverse direction. Repeated physical exercise increases mitochondrial oxidative capacity and alters the utilization of various substrates. Consequently, the amount of lactate produced in trained individuals depends not only on the rate of glycolysis but also on the oxidative phenotype established through mitochondrial adaptation.

Reducing the muscle’s capacity to export lactate in MCT4-knockout mice led to increased intramuscular lactate concentrations but also resulted in a highly interesting metabolic phenotype: enhanced capacity for high-intensity exercise. Increased activity was observed in the glycolytic enzymes hexokinase, glycogen phosphorylase, and pyruvate kinase, along with elevated oxygen consumption using pyruvate as a substrate and increased activity of PDH, isocitrate dehydrogenase, and cytochrome-c oxidase [71].

AMPK, NADH/NAD^+^ balance, redox signaling, and PGC-1α should not be viewed as components of a linear lactate-to-mitokine cascade, but rather as interconnected metabolic nodes through which glycolytic lactate flux and mitochondrial stress signaling may converge and reciprocally reshape one another.

To distinguish experimentally demonstrated mechanisms from indirect associations and hypothetical interactions, Table 2 critically summarizes the available evidence supporting the proposed lactate–mitokine axis. Particular emphasis is placed on the experimental models, the nature of the interventions, and the extent to which the findings establish direct causal relationships between lactate signaling and mitokine responses. This comparison highlights the differences in mechanistic strength among FGF21, GDF15, MOTS-c, and Humanin, while identifying important gaps in the evidence derived from exercise studies. These findings identify AMPK, NAD^+^/NADH redox balance, and PGC-1α as potential points of convergence between lactate metabolism and mitokine-associated signaling. However, activation of shared molecular pathways does not, by itself, establish a direct functional regulatory relationship between lactate and mitokines. Such a relationship would require experimental evidence demonstrating that selective manipulation of lactate availability or signaling alters mitokine production or activity under defined physiological conditions.

## 6. Exercise Modality and Training Status as Modulators of the Lactate–Mitokine Axis

The metabolic signals generated during physical exercise depend on the intensity, duration, modality and degree of training of each individual. All these factors shape the lactate–mitokine axis, customizing glycolytic flow, lactate exposure, ATP turnover, redox perturbation, mitochondrial stress, and endocrine signaling.

An important consideration when comparing exercise modalities is the temporal dissociation between lactate dynamics and endocrine stress responses. Blood lactate concentrations change rapidly during exercise and recovery, whereas circulating mitokines may exhibit delayed or more sustained responses. For example, lactate concentrations peak immediately after high-intensity exercise and subsequently decline during recovery [62], while circulating Humanin may remain elevated for several hours after endurance exercise [56]. FGF21 and GDF15 also respond to exercise-induced metabolic stress, particularly during prolonged endurance exercise, but their temporal profiles may differ depending on the underlying regulatory mechanisms [53,72]. Importantly, changes in circulating mitokine concentrations do not necessarily coincide with changes in tissue expression or secretion. In conclusion, temporal overlap between lactate and mitokine responses should not be assumed, and differences in their kinetics must be considered when interpreting potential interactions within the proposed lactate–mitokine axis.

Differences in blood sampling protocols further complicate comparisons across studies, as measurements obtained immediately after exercise may capture the lactate response but precede delayed changes in circulating mitokines. Later sampling may detect endocrine responses after lactate concentrations have substantially declined. Therefore, the absence of a concurrent lactate–mitokine association at a single sampling time point should not be interpreted as evidence against a potential temporal relationship. Serial measurements during exercise and recovery are needed to characterize these distinct response kinetics.

Beyond exercise modality and intensity, nutritional status, muscle glycogen availability, training history, and participant characteristics may independently influence metabolic and endocrine responses. Fasting and reduced glycogen availability can modify substrate utilization and cellular energy stress, potentially affecting lactate production and stress-responsive signaling pathways. Training adaptations may alter lactate production and clearance, mitochondrial oxidative capacity, and the magnitude of endocrine responses to a given exercise workload. Furthermore, age, sex, adiposity, and metabolic health may contribute to interindividual variability in circulating FGF21 and GDF15 concentrations. These factors should therefore be considered when comparing exercise-induced lactate and mitokine responses across studies, particularly when differences in participant characteristics or metabolic conditions may confound the interpretation of their proposed relationship.

### 6.1. Low- and Moderate-Intensity Continuous Exercise: Sustained Oxidative Metabolism with Limited Lactate Accumulation

Low- and moderate-intensity continuous exercise is generally characterized by predominantly oxidative ATP production and relatively limited blood lactate accumulation, although these responses vary with exercise intensity and individual training status.

In nine young men completing three exercise protocols in a randomized crossover design, 30 min of moderate-intensity continuous cycling at 55% heart-rate reserve (HRR) showed the smallest blood lactate response compared with vigorous continuous exercise at 85% HRR and HIIE consisting of four 30 s all-out Wingate bouts separated by 4 min recovery periods. Blood lactate increased in an intensity-dependent manner, reaching the highest concentrations immediately after HIIE and remaining elevated at 30 and 90 min of recovery, whereas the increase following vigorous continuous exercise was smaller and returned to baseline by 30 min post-exercise [62].

A single 60 min bout of cycling at 65% of VO_2_peak was sufficient to induce measurable changes in skeletal-muscle mitochondrial function. High-resolution respirometry performed on mitochondria obtained from vastus lateralis biopsies showed a 13% increase in lipid/F-linked respiration supported by palmitoyl–carnitine and malate, indicating enhanced capacity for fatty-acid-derived substrate oxidation. N-linked respiration, supported by glutamate and malate and reflecting predominantly Complex I-dependent electron transfer, increased by 17%, while S-linked respiration supported by succinate, which supplies electrons through Complex II via FADH_2_, increased by 14%. Together, these substrate-specific increases indicate that even a single bout of moderate-to-vigorous endurance exercise can acutely enhance mitochondrial oxidative capacity across multiple electron-entry pathways rather than selectively affecting a single respiratory substrate [73].

Repeated moderate-intensity continuous exercise can produce skeletal-muscle mitochondrial remodeling. In ten healthy young adults (five men and five women), four weeks of single-leg cycling training, comprising 12 sessions, were performed using a within-participant design in which one leg was randomly assigned to continuous training and the contralateral leg to interval training. Despite matching absolute and mean exercise intensity, total training time, and training volume between conditions, citrate synthase maximal activity, mitochondrial respiratory-chain protein content, and subsarcolemmal mitochondrial volume increased only after continuous training, whereas no comparable mitochondrial remodeling was observed after interval training [74].

From a training perspective, this type of exercise favors the mitochondrial arm of the network while producing comparatively lower systemic lactate exposure.

### 6.2. HIIT and Sprint Exercise: A Window for Lactate–Mitokine Convergence

HIIT and sprinting are characterized by rapid ATP turnover and the recruitment of glycolytic type II fibers where rapid glycolysis and glycogenolysis occur, which all lead to increased lactate levels. At the same time, repeated high-intensity contractions induce substantial energetic, redox, Ca^2+^, and mitochondrial stress.

In previously untrained men, eight weeks of HIIT (30 s sprint at 85–95% HRmax) reduced peak blood lactate during a standardized high-intensity running test and accelerated post-exercise lactate clearance, with approximately 89% of peak lactate cleared within 30 min compared with 67% before training. These findings indicate that repeated HIIE can substantially remodel subsequent lactate handling [75].

HIIT exercise also triggers the secretion of mitochondria-derived peptides. Muscle biopsies performed after 60 s cycling intervals at a power output corresponding to VO_2_peak revealed an increase in the mitochondria-derived peptide humanin and a transient rise in SHLP2, while SHLP6 levels remained unchanged, suggesting the activation of a mitochondrial stress response [59].

Together, these findings indicate that high-intensity exercise can elicit pronounced lactate responses alongside mitochondrial stress-associated signaling. However, whether these responses are mechanistically coupled or represent parallel adaptations to repeated exercise remains to be established.

### 6.3. Prolonged Endurance Exercise: Cumulative Metabolic Stress and Mitokine Responses

During prolonged submaximal exercise, the variation in lactate concentration is minimal. The increased demand for ATP, the depletion of energy reserves, hepatic metabolic demand, and sustained mitochondrial activity can induce systemic stress signaling.

Four hours of cycling performed by elite triathletes resulted in a 5.3-fold increase in circulating GDF15, while completing a marathon produced an approximately 4.5-fold increase. Thus, the GDF15 response appears to be closely linked to the duration of the effort, rather than merely the type of exercise or the magnitude of glycolytic stress [53]. In marathon runners with a median time of approximately 3.5 h, circulating FGF21 increased approximately 20-fold immediately after the race, while GDF15 increased 4.2-fold. The magnitude of the FGF21 and GDF15 responses was not correlated, suggesting that prolonged endurance exercise can elicit distinct stress-responsive endocrine signals [72].

These findings suggest that prolonged endurance exercise can elicit substantial FGF21 and GDF15 responses despite relatively modest lactate accumulation, highlighting the importance of exercise duration and systemic metabolic stress rather than lactate concentration alone.

### 6.4. Resistance Exercise: Local Glycolytic Stress Within a Mechanically Dominated Signal

Resistance exercise provides a distinct context for examining lactate–mitokine interactions, as local lactate accumulation occurs alongside mechanical loading and contraction-dependent signaling, complicating the attribution of metabolic adaptations specifically to lactate.

By comparison with resistance exercise consisting of four sets at 7RM of leg press and knee extension, endurance exercise increased circulating Humanin by approximately 39% at 30 min, with concentrations remaining elevated 3 h post-exercise, while circulating MOTS-c followed a similar pattern. Both types of effort induced skeletal-muscle transcriptional programs: resistance exercise markedly increased c-Myc and 45S pre-rRNA, consistent with ribosome biogenesis, whereas endurance exercise preferentially increased PGC-1α transcripts [56].

Increased lactate availability during resistance exercise, however, does not appear to be sufficient to amplify the anabolic response. Intravenous administration of sodium lactate during one-legged resistance exercise increased circulating lactate from approximately 3 to 7 mmol·L^−1^. Resistance exercise activated mTOR/S6K1 and ERK signaling and stimulated muscle protein synthesis but experimentally increasing lactate availability did not further enhance these responses [25].

During resistance exercise, the lactate–myokine axis is modulated according to the specific type of exercise. Glycolytic and lactate responses occur in the context of mechanical loading and local anabolic remodeling, whereas responses involving mitochondria-derived peptides are less pronounced than those observed during endurance training or HIIE. In this context, lactate serves merely as one component of resistance exercise rather than an independent driver of the adaptive response.

### 6.5. Training Status: Is the Lactate–Mitokine Axis Trainable?

The type of exercise performed serves merely as an acute stimulus acting upon the individual’s pre-existing metabolic phenotype. Repeated training increases mitochondrial respiratory capacity and alters substrate utilization, resulting in lactate levels and mitochondrial stress patterns that differ from those observed in untrained individuals (see Table 3).

Recent longitudinal evidence demonstrates that lactate handling itself is trainable. The training modality can reshape the systemic lactate response to an identical exercise challenge. In previously untrained young men, eight weeks of HIIT or MICT, performed three times per week, significantly reduced the blood lactate response to a standardized 1 min high-intensity running test at 20 km·h^−1^. HIIT consisted of repeated 30 s running bouts at 85–95% of maximal heart rate interspersed with 3 min active recovery, whereas MICT involved continuous running at 65–75% of maximal heart rate. After eight weeks, peak blood lactate decreased both in the HIIT and the MICT groups but the decrease was much more pronounced in the HIIT group [75]. Importantly, lower blood lactate concentrations following endurance training should not be interpreted as direct evidence of enhanced lactate clearance. Circulating lactate levels reflect the dynamic balance between lactate production, oxidation, and intertissue exchange, all of which may be modified by training-induced metabolic adaptations. Therefore, without direct measurements of lactate appearance and disappearance rates, the relative contributions of these processes cannot be established.

Repeated exercise also remodels skeletal-muscle oxidative machinery. In young and older adults, six weeks of aerobic cycling combining continuous and interval sessions increased citrate synthase activity by 45% and 31%, respectively, and mitochondrial respiratory-chain complex I–IV activities by 40–163%. Importantly, after eight weeks of deconditioning, citrate synthase and several respiratory-complex activities declined more markedly in older participants, indicating that mitochondrial adaptations remain inducible with aging but may be less well maintained during inactivity [76].

Repeated interval exercise induces substantial functional remodeling of skeletal-muscle mitochondria. Six weeks of single-leg interval training performed three times per week increased maximally coupled mitochondrial respiration by 27.9% and uncoupled respiration by 33.2%. Training also increased fatty-acid oxidation and increased citrate synthase activity [77].

Although longitudinal studies demonstrate training-induced adaptations in circulating lactate responses and skeletal-muscle mitochondrial function [74,76,77], evidence for coordinated adaptations between lactate metabolism and mitokine production remains limited. Most available investigations have examined these responses separately, without simultaneously assessing lactate kinetics and mitokine expression or secretion throughout a training intervention. In conclusion, parallel changes in lactate metabolism and mitochondrial stress signaling cannot be interpreted as evidence of a direct regulatory relationship. Longitudinal studies combining lactate kinetic measurements with repeated assessments of FGF21, GDF15, MOTS-c, and Humanin are needed to determine whether these responses undergo coordinated adaptations or represent independent consequences of repeated exercise.

## 7. Translational Perspectives and Potential Applications of the Lactate–Mitokine Axis

The potential translational value of the lactate–mitokine axis lies in integrating metabolic and mitochondrial stress responses to better characterize exercise adaptation. Although blood lactate is routinely used to assess exercise intensity, its concentration reflects the balance between production, transport, oxidation, and clearance rather than signaling activity alone. Combining lactate kinetics with circulating FGF21, GDF15, MOTS-c, and Humanin measurements could provide additional information about metabolic responsiveness and mitochondrial stress. However, the interpretation of circulating FGF21, GDF15, MOTS-c, and Humanin as biomarkers of exercise-induced mitochondrial stress is constrained by several biological and analytical limitations. FGF21 and GDF15 are not specific to exercise-induced mitochondrial dysfunction and may originate from multiple tissues in response to metabolic, inflammatory, or other cellular stressors. Their circulating concentrations are also influenced by nutritional status, metabolic health, age, and other individual characteristics, complicating the attribution of observed changes to exercise alone. For MOTS-c and Humanin, the relationship between circulating concentrations, tissue-specific production, and mitochondrial adaptive responses remains insufficiently characterized. Furthermore, differences in assay specificity, sensitivity, calibration, and reproducibility, particularly for mitochondrial-derived peptides, may limit comparability across studies. To conclude, standardized sampling conditions and analytically validated methods are essential before these molecules can be reliably interpreted as biomarkers of exercise adaptation. Beyond exercise monitoring, this framework may have implications for conditions associated with impaired mitochondrial function and metabolic flexibility, including aging, obesity, insulin resistance, and sarcopenia. Nevertheless, the available evidence does not establish that altered lactate–mitokine interactions contribute causally to these conditions or that targeting this proposed axis improves clinical outcomes. Similarly, although lactate administration and selected mitokines have demonstrated metabolic effects in experimental models, these interventions cannot currently be considered substitutes for exercise.

Future investigations should prioritize controlled human studies combining standardized exercise interventions, lactate kinetic measurements, and serial assessments of circulating and tissue-specific mitokines. Where feasible, experimental manipulation of lactate availability or signaling should be incorporated to distinguish direct regulatory effects from parallel responses to exercise-induced metabolic stress. Longitudinal training studies are also needed to determine whether changes in lactate–mitokine profiles predict mitochondrial adaptation, metabolic flexibility, or functional improvements independently of established exercise biomarkers.

At present, the combined assessment of lactate kinetics and circulating mitokines remains a research-oriented approach rather than a validated biomarker strategy. Although individual exercise-induced changes in lactate, FGF21, GDF15, MOTS-c, and Humanin have been reported, evidence demonstrating that their combined measurement provides additional physiological or predictive information remains insufficient. Before broader application, future studies should establish standardized exercise and sampling protocols, validate analytical methods, account for biological and interindividual variability, and determine the reproducibility of these responses. Importantly, longitudinal investigations should assess whether integrated lactate–mitokine profiles predict training adaptations or clinically relevant outcomes beyond established measures of exercise capacity and metabolic function.

Thus, the lactate–mitokine axis should currently be regarded as a hypothesis-generating framework with potential applications in metabolic phenotyping and exercise research, rather than a validated approach to exercise prescription or therapeutic intervention.

## 8. Conclusions

Adaptation to physical effort is achieved through the integration of various metabolic mechanisms. Lactate and mitochondrial stress both result from increased energy demands and possess paracrine signaling potential. Lactate plays multiple roles: serving as an oxidative substrate (linked to the NAD+/NADH ratio) and facilitating inter-organ carbon shuttling, while also acting as a potential HCAR1 signaling molecule or mediating lactylation. Exercise-induced mitochondrial stress activates energy and redox pathways but also triggers the release of mitokines such as FGF21, GDF15, MOTS-c, and Humanin. Glycolytic/lactate signaling and mitochondrial stress responses converge at key regulatory nodes, most notably AMPK, p38 MAPK, NAD^+^/NADH, SIRT1, and PGC-1α.

The proposed lactate–mitokine axis should therefore be regarded as a conceptual framework linking glycolytic metabolism and mitochondrial stress signaling, rather than an established bidirectional regulatory pathway. Although these processes converge on shared metabolic and signaling mechanisms, such convergence does not necessarily imply direct regulatory coupling. Experimental findings involving lactate-dependent FGF21 regulation provide evidence of a potential interaction in specific tissue and experimental contexts, but their relevance to systemic endocrine responses during human exercise remains uncertain. Moreover, direct lactate-dependent regulation of GDF15, MOTS-c, and Humanin has not been sufficiently demonstrated.

Several fundamental questions remain unresolved: whether physiological changes in lactate availability directly regulate mitokine expression or secretion during exercise; whether mitokine signaling reciprocally influences lactate production, transport, or oxidation; and whether these interactions undergo coordinated adaptations following repeated training. Addressing these questions will require targeted manipulation of lactate signaling, tissue-specific mechanistic investigations, and longitudinal human studies integrating lactate kinetics with mitokine measurements. Until such evidence becomes available, the lactate–mitokine axis represents a hypothesis-generating model for investigating metabolic and mitochondrial communication during exercise, rather than a physiologically validated regulatory system.

## Figures and Tables

**Table 2:** Experimental evidence, mechanistic strength, and unresolved causal relationships within the proposed lactate–mitokine axis.

Proposed Interaction	Experimental Model/Intervention	Study Design and Evidence Type	Main Experimental Findings	Evidence Interpretation and Limitations	Ref.
Lactate–FGF21 (systemic association)	Human crossover study; 9 healthy young men (24.0 ± 0.4 years); CTRL, MICE (55% HRR), VICE (85% HRR), and HIIE (4 × 30 s all-out cycling, 4 min recovery); blood sampling before, immediately after, and 30 and 90 min after exercise.	Human interventional exercise study; observational lactate–FGF21 association	HIIE increased serum FGF21 immediately after exercise compared with CTRL. FGF21 AUC was higher after HIIE than CTRL and MICE. Changes in blood lactate positively correlated with changes in FGF21.	Human experimental evidence for exercise intensity-dependent FGF21 responses and lactate–FGF21 association. Limitations: Lactate was not independently manipulated; correlation does not establish direct lactate-mediated FGF21 secretion. Small sample (n = 9); healthy young men only.	[62]
Lactate–FGF21 (central GPR81/p38-MAPK signaling)	Male Wistar rats; intracerebroventricular lactate or FGF21 administration; acute endurance exercise + inhibition of brain lactate transport (4-CIN)/systemic lactate production (DCA); GPR81/p38-MAPK inhibition.	Animal interventional study; targeted pharmacological manipulation and tissue-specific mechanistic evidence.	Exercise increased hypothalamic and CSF FGF21 Central lactate reproduced FGF21 induction via GPR81/p38-MAPK inhibition of brain lactate transport attenuated FGF21 induction.	Direct, tissue-specific mechanistic evidence for lactate-dependent hypothalamic FGF21 regulation in rats. Limitations: FGF21 regulation or during human exercise not established.	[63]
Mitochondrial stress–FGF21/GDF15 signaling	LKO mice combined with global Fgf21/Gdf15 knockout.	Animal genetic intervention; causal evidence for mitokine-mediated metabolic effects.	Increased hepatic mitochondrial stress and FGF21 and GDF15 expression. Increased circulating FGF21-produced glucose clearance, adipose thermogenesis, and energy expenditure. GDF15 regulated body/fat mass and protected against hepatic steatosis.	Endocrine functions of FGF21 and GDF15 during mitochondrial stress. Limitations: Genetic mitochondrial dysfunction is not equivalent to exercise; direct lactate-dependent regulation not investigated.	[51]
Muscle mitochondrial stress–FGF21 signaling	UCP1-transgenic mice with skeletal muscle mitochondrial uncoupling, crossed with Fgf21-knockout mice; LFD/HFD.	Animal genetic intervention; FGF21-dependent endocrine effects.	FGF21 deletion abolished mitochondrial stress-induced browning of WAT but did not prevent improved glycemic control, obesity resistance, hepatic lipid protection, or muscle mitohormetic adaptations.	Selective endocrine role of FGF21 in adipose remodeling. Limitations: Genetic mouse model lactate-dependent regulation not examined.	[52]
Exercise-induced metabolic stress–GDF15 signaling	Human endurance and resistance exercise studies; exhaustive treadmill running in wild-type and GFRAL-knockout mice; pharmacological GDF15 administration.	Human exercise studies and animal genetic/pharmacological interventions; mixed experimental evidence.	Prolonged endurance exercise increased circulating GDF15 4–5-fold in humans and mice. Pharmacological GDF15 suppressed voluntary running through GFRAL. Exercise-induced endogenous GDF15 did not mediate post-exercise reductions in running activity or food intake.	GDF15 as an exercise-responsive stress signal, but not as a demonstrated mediator of exercise-induced behavioral changes. Limitations: Lactate regulation of GDF15 not investigated; direct lactate–GDF15 interaction unproven.	[53]
Exercise-induced metabolic stress–GDF15 signaling	Human studies (2 h cycling at 60% VO_2_max); arterial–hepatic venous sampling glucagon infusion, and pancreatic clamp during exercise. Acute treadmill exercise in mice.	Human physiological and hormonal interventions; complementary animal exercise experiments.	Exercise increased circulating GDF15 and stimulated hepato-splanchnic release. Increasing the glucagon-to-insulin ratio elevated plasma GDF15 approximately 2.7-fold, pancreatic clamp blunted the exercise-induced GDF15 response. Exercise increased GDF15 mRNA in mouse liver, skeletal muscle, and adipose tissue.	Hormonal regulation of exercise-induced GDF15, predominantly hepato-splanchnic contribution. Limitations: Lactate signaling not experimentally manipulated; direct lactate–GDF15 regulatory relationship unproven.	[54]
Exercise-induced metabolic stress–GDF15 response	Human randomized controlled trial, (90 inactive adults with overweight/obesity). Acute cycling at 60% VO_2_peak and 6-month moderate- or vigorous-intensity exercise training (5 days/week).	Human randomized controlled exercise trial; acute and longitudinal responses.	Increased plasma GDF15 by 18% immediately and 32% at 60 min post-exercise. Six months of training did not significantly alter fasting GDF15 or its acute exercise response.	Acute exercise-induced GDF15 response. Limitations: Lactate-dependent signaling not directly investigated; causal lactate–GDF15 interaction not established.	[55]
Mitochondrial stress–GDF15 signaling and systemic metabolic adaptation	Ucp1-transgenic mice crossed with Gdf15-knockout mice; FCCP-treated C2C12 myotubes.	Animal genetic intervention and cellular pharmacological model; mechanistic evidence.	Mitochondrial stress induced skeletal muscle GDF15 expression and secretion. Gdf15 deletion abolished stress-associated improvements in systemic insulin sensitivity, adipose tissue browning, and metabolic flexibility.	GDF15-mediated endocrine responses to mitochondrial dysfunction in mice. Limitations: Model involves chronic mitochondrial uncoupling rather than physiological exercise. Lactate-dependent regulation of GDF15 not investigated.	[50]
Exercise-induced Humanin and MOTS-c responses; potential association with lactate signaling	Human study (n = 30): Acute endurance exercise (45 min cycling at 70% estimated VO_2_max), resistance exercise (4 sets at 7RM), or control. Plasma and muscle samples before, 30 min, and 3 h after exercise.	Human controlled acute exercise study; observational mitokine responses.	Endurance exercise increased circulating Humanin by ~39% at 30 min, with sustained elevation at 3 h. Circulating MOTS-c showed a nonsignificant upward trend. Skeletal muscle MOTS-c did not change significantly.	acute exercise-induced Humanin response, Limitations: lactate-signaling not assessed; a causal lactate–Humanin or lactate–MOTS-c interaction cannot be established.	[56]
Exercise-induced Humanin response; hypothetical involvement of lactate signaling	Human study: Acute HIIT (10 × 60 s cycling at VO_2_peak power, separated by 75 s low-intensity recovery). Ex vivo electrically stimulated mouse EDL muscle.	Human exercise intervention and ex vivo animal muscle contraction experiments.	Increased circulating and skeletal muscle Humanin. Isolated muscle contraction increased. Short-term HIIT did not significantly alter the acute Humanin response.	Acute exercise and muscle contraction regulate Humanin abundance. Limitations: Lactate-dependent signaling not experimentally investigated; hypothetical causal lactate–Humanin interaction.	[59]
Lactate-induced modulation of mitochondrial respiratory function	Male C57BL/6J mice; normal or HFD; i.p. sodium L-lactate (initially 1.5 g/kg, subsequently 2 g/kg, 5 days/week for 8 weeks).	Animal pharmacological/metabolic intervention; direct lactate administration.	Lactate enhanced skeletal muscle fatty acid oxidation, citrate synthase activity, and mitochondrial respiration supported by fatty acid-derived substrates in high-fat-diet-fed mice. Increased UCP3 abundance and reduced respiratory coupling were also observed.	Supports lactate-induced modulation of skeletal muscle oxidative metabolism under high-fat diet conditions. However, the study did not establish direct lactate–mitokine coupling, and the findings cannot be directly extrapolated to physiological exercise-induced lactate responses.	[64]
Exercise-induced lactate production and AMPK signaling; modulation by PGC-1α	Male mice, 6-week endurance skeletal muscle-specific PGC-1α overexpression. Acute exercise at increasing intensities.	Animal exercise and genetic intervention; associative lactate–AMPK findings.	Endurance training and PGC-1α overexpression reduced estimated lactate production at the same absolute exercise intensity. At 40 m/min, estimated lactate production and AMPK phosphorylation increased, accompanied by reduced muscle glycogen. No significant changes in p38 MAPK or CaMKII phosphorylation were detected.	Supports lactate-induced modulation of skeletal muscle oxidative metabolism under high-fat diet conditions. However, the study did not establish direct lactate–mitokine coupling, and the findings cannot be directly extrapolated to physiological exercise-induced lactate responses.	[65]
Exercise-induced Humanin response and potential lactate–mitokine coupling	Ten healthy, untrained men acute HIIT (10 × 60 s cycling at VO_2_peak power output). Isolated mouse EDL muscle contractions ex vivo.	Human exercise intervention and ex vivo animal muscle contraction experiments.	Acute exercise increased circulating Humanin and relative skeletal muscle Humanin abundance. Isolated muscle contractions increased Humanin approximately 4×. Short-term HIIT did not significantly modify the acute Humanin response.	Direct experimental evidence of exercise-responsive Humanin regulation. Limitations: Lactate–Humanin coupling was not directly investigated; small, male-only human cohort.	[57]
MOTS-c–AMPK/PGC-1α signaling and mitochondrial adaptation	PGC-1α and AMPKα1/α2 knockout mice; MOTS-c administration (5 mg/kg, 5 days/week for 4 weeks); acute exercise and voluntary wheel running. Human skeletal muscle cells treated with AICAR,	Animal genetic/pharmacological interventions and human cellular experiments; mechanistic evidence,	MOTS-c enhanced mitochondrial respiration and reduced ROS emission in mouse skeletal muscle, with these effects dependent on PGC-1α and partly on AMPK. Exercise increased muscle MOTS-c expression, while AICAR increased MOTS-c and PGC-1α mRNA in human myocytes.	MOTS-c-mediated mitochondrial adaptation through PGC-1α/AMPK, but not for lactate-dependent MOTS-c regulation. Limitations: No lactate manipulation; no causal lactate–MOTS-c interaction	[66]

Note: The mechanistic strength of the evidence was qualitatively evaluated according to the experimental model (human, animal, or cellular), study design, use of targeted interventions, and ability to establish causal relationships. Observational associations were distinguished from experimentally demonstrated effects, while findings involving shared metabolic or signaling pathways were considered indirect support unless a specific lactate–mitokine regulatory interaction was experimentally demonstrated. Evidence obtained in animal or cellular models was interpreted within its experimental context and was not directly extrapolated to human exercise physiology.

**Table 3:** Exercise load-dependent lactate and mitokine responses: acute effects, chronic adaptations, and evidence gaps.

Exercise Load/Modality	Acute Lactate Response	Acute Mitokine Response	Chronic Training Adaptation	Evidence Interpretation	Ref.
Moderate-intensity continuous exercise (MICE)	Generally lower lactate accumulation than high-intensity exercise	Acute GDF15 increases of 18% immediately and 32% at 60 min after moderate cycling	Six months of moderate- or vigorous-intensity training did not significantly alter basal GDF15 or its acute exercise response	Human evidence for acute GDF15 responsiveness, but no established lactate-dependent regulation	[55,62]
Vigorous-intensity continuous exercise (VICE)	Greater glycolytic demand; lactate responses depend on workload and training status	FGF21 responses assessed alongside lactate in a human crossover study	Mitokine-specific chronic adaptations not established in the cited comparison	Human comparative evidence; direct lactate–FGF21 causality unproven	[62]
HIIE/HIIT	Substantial transient lactate accumulation	Increased FGF21 exposure after HIIE; exercise-associated Humanin responses reported	Repeated HIIT improves mitochondrial respiration and modifies lactate responses to standardized exercise	Human evidence for distinct acute responses and training adaptations, but not for causal lactate–mitokine coupling	[41,59,62,75]
Prolonged endurance exercise	Lactate concentration may remain relatively modest despite sustained metabolic turnover	Marked increases in circulating GDF15 and FGF21 after prolonged cycling or marathon running	Direct longitudinal evidence linking lactate kinetics with mitokine adaptation is insufficient	Duration-associated endocrine stress responses; independent effects of duration and intensity remain unresolved	[53,72]
Resistance exercise	Local glycolytic stress and variable systemic lactate accumulation	Humanin and MOTS-c responses vary by protocol; endurance and resistance responses are not equivalent	Chronic lactate-dependent mitokine regulation not established	Mechanical and metabolic stimuli cannot be readily separated	[25,56,59]
Repeated endurance or interval training	Lower lactate response to a standardized absolute workload after training	Longitudinal mitokine responses remain insufficiently characterized	Improved oxidative capacity, mitochondrial respiration and lactate handling	Chronic metabolic adaptation established; corresponding mitokine-mediated adaptation unproven	[75,76,77]

Note: Acute responses refer to changes occurring during exercise or within the immediate post-exercise recovery period, whereas chronic adaptations reflect changes following repeated training interventions. Exercise intensity, session duration, and accumulated training volume are interrelated variables that have not been independently manipulated in most available studies. Furthermore, evidence of exercise-induced changes in lactate and mitokine concentrations does not establish a direct causal relationship between these responses.

## Data Availability

No new data were created or analyzed in this study. Data sharing is not applicable to this article.

## Abbreviations

The following abbreviations are used in this manuscript:

AARS1	alanyl-tRNA synthetase 1
ACC	acetyl-CoA carboxylase
ACT	acylcarnitine translocase
ADP	adenosine diphosphate
Akt	protein kinase B
AMPc	cyclic adenosine monophosphate
AMPK	adenosine monophosphate-activated protein kinase
ATF4	activating transcription factor 4
ATP	adenosine triphosphate
ATPase	adenosine triphosphatase
BCAAs	branched-chain amino acids
BCAT	branched-chain amino acid aminotransferase
BCKA	branched-chain α-keto acid
BCKDH	branched-chain α-keto acid dehydrogenase
CK	creatine kinase
CNS	central nervous system
COX	cytochrome c oxidase
CPT1/2	carnitine palmitoyltransferase ½
Cr	creatine
CREB-S133	cyclic adenosine monophosphate response element-binding protein
CS	citrate synthase
CSF	cerebrospinal fluid
DGAT	diacylglycerol acyltransferase
ERK	extracellular signal-regulated kinase
ETC	electron transport chain
FAT/CD36	fatty acid translocase/cluster of differentiation 36
FFA	free fatty acids
FGF21	fibroblast growth factor 21
FGFR1c	fibroblast growth factor receptor 1c
G-1-P	glucose-1-phosphate
G-6-P	glucose-6-phosphate
GA3PDH	glyceraldehyde-3-phosphate dehydrogenase
GDF15	growth differentiation factor 15
GFRAL	GDNF family receptor alpha-like
GLUT1/4	glucose transporter ¼
GPAT	glycerol-3-phosphate acyltransferase
GPR81	G protein-coupled receptor 81
GPx	glutathione peroxidase
HCAR1	hydroxycarboxylic acid receptor 1
HFD	high-fat-diet
HIF-1α	hypoxia-inducible factor 1-alpha
HIIE	high-intensity interval exercise
HIIT	high-intensity interval training
HK	hexokinase
HRR	heart-rate reserve HRR
HS	hormone-sensitive lipase
HSF1	heat shock factor 1
Ile	isoleucine
ISR	integrated stress response
JNK	c-Jun N-terminal kinase
LAT1	L-type Amino Acid Transporter 1
LCAD	long-chain acyl-CoA dehydrogenase
LDH	lactate dehydrogenase
Leu	Leucine
LT	lactate threshold
MCT	monocarboxylate transporter
MD	mitochondrial dysfunction
MDP	mitochondrial-derived peptide
MG lipase	monoglyceride lipase
MICT	moderate-intensity continuous training
MLSS	maximal lactate steady state
MOTS-c	mitochondrial ORF of the 12S rRNA type-c
mtCK	mitochondrial creatine kinase
mTOR	mechanistic target of rapamycin
mTORC1	mechanistic target of rapamycin complex 1
mtROS	mitochondrial reactive oxygen species
NAD^+^	nicotinamide adenine dinucleotide
NADH + H^+^	reduced nicotinamide adenine dinucleotide
OXPHOS	oxidative phosphorylation
p38 MAPK	p38 mitogen-activated protein kinase
PCr	phosphocreatine
PDH	pyruvate dehydrogenase
PGC-1α	peroxisome proliferator-activated receptor gamma coactivator 1-alpha
Pi	inorganic phosphate
PI3K	phosphoinositide 3-kinase
PKA	protein kinaseA
RET	rearranged during transfection receptor tyrosine kinase
S6K1	ribosomal protein S6 kinase 1
SCAD	short-chain acyl-CoA dehydrogenase
SIRT6	sirtuin 6
SOD2	superoxide dismutase 2
SR	sarcoplasmic reticulum
TCA	tricarboxylic acid
TG	triglycerides
Val	valine
VEGF	vascular endothelial growth factor
WAT	white adipose tissue
α-KG	α-ketoglutarate
